# Recyclable Covalent Adaptable Polystyrene Networks Using Boronates and TetraAzaADamantanes

**DOI:** 10.1021/acsapm.4c01633

**Published:** 2024-06-29

**Authors:** Simon van Hurne, Sagar Kumar Raut, Maarten Marinus Johannes Smulders

**Affiliations:** Laboratory of Organic Chemistry, Wageningen University, Stippeneng 4, 6708 WE Wageningen, The Netherlands

**Keywords:** vitrimer, covalent adaptable network, boronic ester, polystyrene, recycling

## Abstract

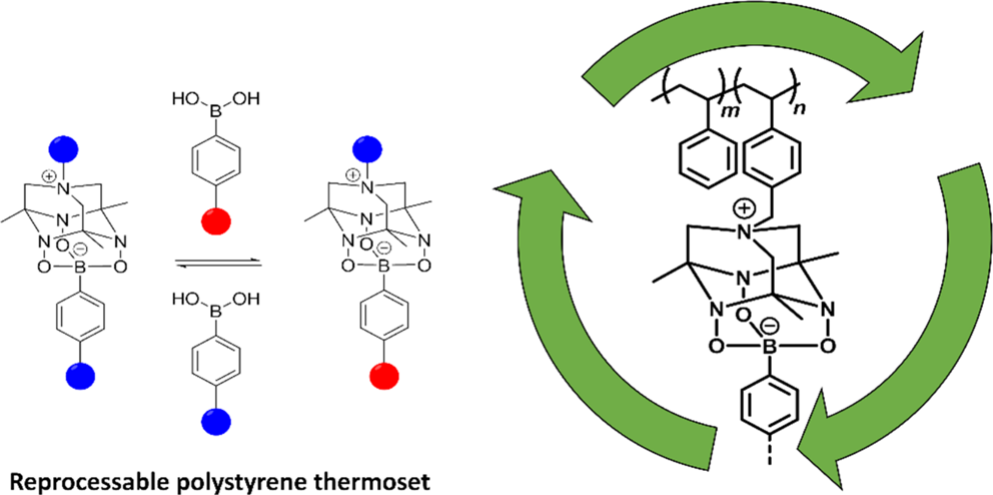

With an ever-increasing annual production of polymers and the accumulation of polymer waste leading to progressively adverse environmental consequences, it has become important that all polymers can be efficiently recycled at the end of their life cycle. Especially thermosets are intrinsically difficult to recycle because of their permanent covalent cross-links. A possible solution is to switch from using thermosets to covalent adaptable networks, sparking the rapid development of novel dynamic covalent chemistries and derived polymer materials. Next to development of these innovative polymer materials, there is also an evident advantage of merging the virtues of covalent adaptable networks with the proven material properties of widely used commodity plastics, by introducing dynamic covalent bonds in these original thermoplastic materials to obtain recyclable thermosets. Here we report the synthesis and characterization of a polystyrene polymer, functionalized with TetraAzaADamantanes and cross-linked with dynamic covalent boronic esters. The material properties were characterized for different degrees of cross-linking. The materials showed good solvent resistance with a high remaining insoluble fraction. In line with the typical behavior of traditional covalent adaptable networks, the prepared polystyrene-based boronate-TetraAzaADamantane materials were able to undergo stress relaxation. The material relaxation was also shown to be tunable by mixing with an acid catalyst. Lastly, the materials could be recycled at least 2 times.

## Introduction

In modern times, polymers have become an important material for society because of their versatile use and low production costs.^1^ Because of this, annual polymer production continues to rise every year. This increasing polymer production also leads to increasing polymer waste being produced each year. When that polymer waste ends up in the environment, it leads to a negative environmental impact. This makes the recycling of polymers of increasing importance.^2,3^ However, the degree to which a polymer can be recycled strongly depends on its structure. Thermoplastics, generally composed of linear polymer chains, can be recycled relatively easily. Their more robust thermoset counterparts, on the other hand, are inherently highly difficult or even impossible to recycle due to their permanent cross-links.^4−7^

Over the last few decades, covalent adaptable networks (CANs) have grown in interest as a means of tackling the buildup of polymer waste,^8−11^ while at the same time imbuing novel (dynamic) properties in polymeric materials.^12,13^ CANs are networks that are covalently cross-linked using reversible covalent bonds. The exchange between those reversible covalent bonds gives CANs access to the strength of permanently cross-linked thermosets, while at the same time retaining the reprocessability of non-cross-linked thermoplastics.^14,15^ One of the most interesting properties that covalent adaptable networks have is their ability to heal after damage. This can extend the useful lifetime of such a material, thus reducing the need for replacements.

CANs operate on the principle of dynamic covalent chemistry,^16,17^ where covalent bonds can exchange over time, as opposed to supramolecular chemistry,^18^ where noncovalent bonds can exchange. Examples of dynamic covalent bonds used in CANs are the Diels–Alder reaction,^19,20^ N,S-acetals,^21^ nucleophilic transalkylation reactions,^22^ imines,^23−25^ disulfides,^26−28^ esters,^29,30^ vinylogous urethanes,^31,32^ diketoamines,^33,34^ acylsemicarbazides,^35,36^ and boronic esters.^37−40^ In this study, we chose to use the well-known boronic ester bond, on which we reported previously,^41,42^ for the formation of dynamically cross-linked networks.^43,44^

Boronic esters-containing networks can be prepared via a condensation reaction between bifunctional boronic acids and polyols, such as sugar or poly(vinyl alcohol).^45,46^ Generally, these boronic networks form (hydro)gels and, thus, have low structural integrity and are prone to tearing. However, they exhibit low toxicity due to the biocompatibility of boronic acids, allowing for potential usage in biomedical applications.^47−49^

An elegant possibility to improve the properties of the conventional boronic ester bond is to replace the typical diols with the strong and rigid triol binding of the TetraAzaADamantanes (TAADs).^50−52^ Previously, we investigated the possibility of introducing such TAAD moiety in covalent adaptable networks with boronic acids by using a small linker, resulting in the formation of boronate-TAAD soft rubbers.^41,42^ These rubbers displayed a high healing capacity. However, the resulting soft rubbers might not be suitable for more structural end product applications. This prompted us to look toward introducing boronate-TAAD linkages in established commodity polymers. The process of turning established thermoplastics into dynamically cross-linked CANs can help facilitate the transition toward circular plastics, since the industry is already familiar with the handling of those materials.^53−56^ For our commodity plastic, we chose to use polystyrene, since it is a well-known and widely used plastic ranging from food packaging to components in the automotive industry. In this, our aim was not to make conventional polystyrene recyclable but to make recyclable thermosets (cross-linked polymers) from an established industrially relevant polymer (polystyrene) backbone. Several groups have investigated dynamically cross-linked networks containing styrene moieties. Most of these used styrene–butadiene rubber (SBR) with roughly only 15–19% styrene in the main polymer chain as a platform for cross-linking.^57−60^ In contrast, there are only a few studies that focused on a higher styrene content in their polymers.^25,61−63^ While this did result in recyclable cross-linked polystyrene materials, most of these examples still relied on mixing in nonstyrenic monomers, or used environmentally unfriendly components (e.g., diisocyanates or copper(I)), which could impact the ultimate applicability of such materials.

**Figure 1:**
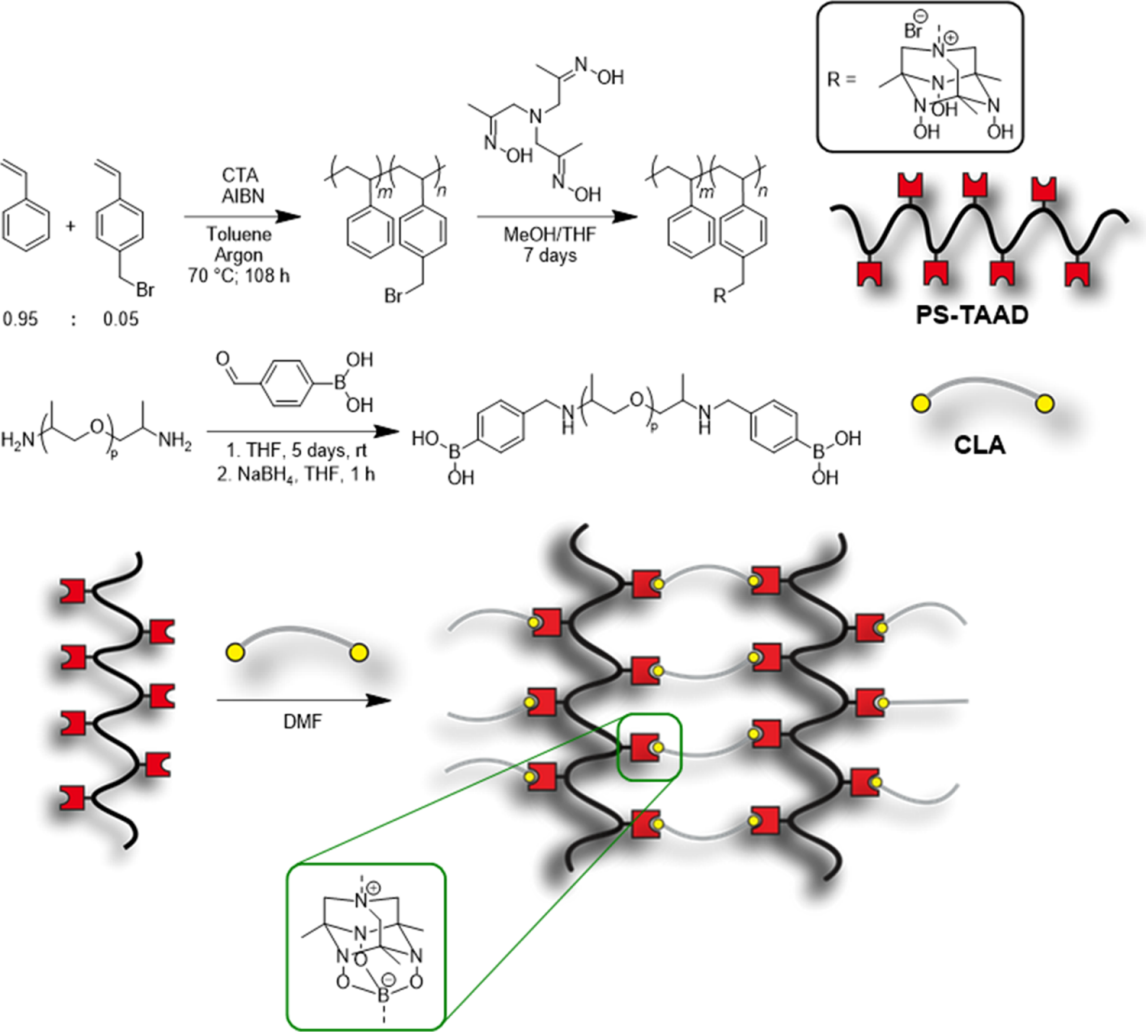
Synthesis of the polystyrene modified with TetraAzaADamantanes, named PS-TAAD, and the polypropylene glycol end-capped with phenylboronic acid, named cross-linking agent (CLA). A schematic representation of the cross-linked network is shown below.

Building on this early work, our aim was to maintain the polystyrene character of the final polymer (network) to a high degree while at the same time incorporating the dynamic bonds (and corresponding dynamic network properties) inside the polymer, to ultimately arrive at polystyrene-based covalent adaptable networks. The former objective was realized by synthesizing (and later functionalizing) a polymer whose backbone is fully composed of styrene monomers, while the latter objective was achieved by integration of the boronate-TAAD linkages (Figure 1).

## Results and Discussion

In order to introduce dynamic covalent bonds in polystyrene, first, 4-vinylbenzyl bromide was copolymerized with styrene (in a molar ratio of 5 to 95, respectively), to create a prepolymer of poly(styrene-*co*-4-vinylbenzyl bromide) using a RAFT polymerization under argon at 70 °C (Figure 1). The 4-vinylbenzyl bromide monomer was prepared by halide exchange from 4-vinylbenzyl chloride with 98% yield. The remaining 2% 4-vinylbenzyl chloride present in the feed was included in the molar ratio of the monomers (see the Supporting Information for full synthetic details and characterization). The poly(styrene-*co*-4-vinylbenzyl bromide) was characterized by gel permeation chromatography (GPC) and nuclear magnetic resonance (NMR). The copolymer had an *M_n_* of 86 kDa (as determined by GPC, calibrated against polystyrene) and 5% incorporated benzyl bromide (as determined by NMR, see the Supporting Information, Figure S3 and Table S1). Subsequently, the introduced benzyl bromide was then used for the attachment of the TetraAzaADamantane (TAAD) moieties via nitrogen quaternization (Figure 1). The incorporation of the TAAD moiety was determined by using NMR to compare the integral value of the TAAD–CH_3_ groups to that of the benzyl groups of the polymer backbone. This revealed quantitative conversion of the bromide end groups into the TAAD moieties. Any remaining 4-vinylbenzyl chloride would also have been converted during the functionalization, albeit at a slower rate. The functionalized polymer was not soluble in toluene, chloroform, THF, or acetone, thus confirming that the quaternization was successful (and, unfortunately, hampering characterization by GPC). Gratifyingly, the increase in molecular weight (and concomitant change in polarity) could be observed by (uncalibrated) DOSY (see Supporting Information, Figures S4 and S6), as indicated by a decrease in the diffusion coefficient of the polymer. Given the large difference in polarity of the two polymers, we refrained from directly comparing the molecular weight by converting the observed diffusion coefficient into a hydrodynamic volume and the corresponding molecular weight. Instead, we determined the molecular weight by NMR: based on the degree of TAAD functionalization (determined by NMR, see Supporting Information, Figure S5), since no change in the length of the polymer was expected. We calculated the increase in molecular weight from the bromide-containing polystyrene polymer (with a known molecular weight of 86 kDa), which resulted in a functionalized polymer named PS-TAAD with an *M_n_* of 96 kDa, with 5% TAAD incorporation. For comparison, a shorter PS-TAAD, with an *M_n_* of only 8.5 kDa, was also prepared and tested (see the Supporting Information for synthetic details and characterization). However, samples prepared with this shorter PS-TAAD were found to have reprocessing issues, and thus this study focused primarily on the longer PS-TAAD. Finally, the bifunctional boronic acid cross-linking agent (CLA) was prepared by reacting 4-formylphenylboronic acid onto amine-terminated PPG and reducing the formed imine with NaBH_4_ (following our previously published procedure).^41,42^

Once the two components were synthesized, they were mixed in different molar ratios relative to the functional groups to prepare a series of materials with different degrees of cross-linking: 25, 50, 75, and 100%, (to clarify, in case of the 50% sample, this meant that within the material there was one boronic acid group per two TAAD moieties). The materials were prepared by dissolving the PS-TAAD in DMF and the CLA in methanol and mixing the two components by vortexing, before casting them in a silicone mold (see also the Supporting Information for further details on material preparation). The solvent was removed by drying the samples for 2 days in an oven at 70 °C, followed by a night in a vacuum oven at 50 °C. This resulted in the formation of yellow-brownish materials.

**Figure 2:**
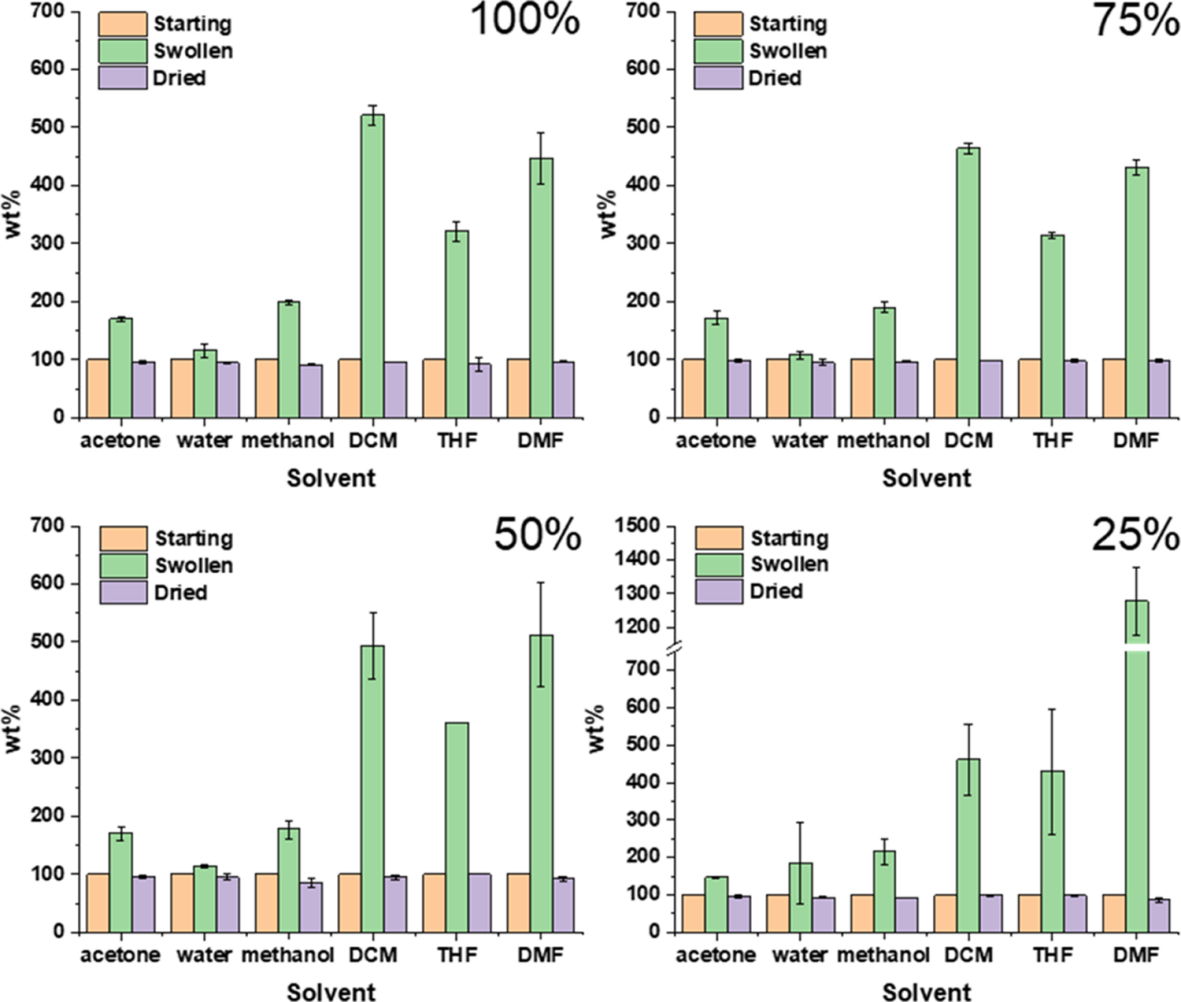
Solvent study of the materials with different degrees of cross-linking. Both the swelling and the remaining fraction are denoted as a weight percentage (wt %), normalized to the starting weight. All measurements were performed *in duplo*.

To determine if the formed materials really form a cross-linked network, the samples with different cross-link degrees were first of all subjected to a solubility (swelling) study in which the materials were soaked in an excess of solvent overnight to determine the swelling behavior and the remaining weight percentage after drying in a vacuum oven (Figure 2). The solvents were chosen to cover a broad range of different well-known and commonly used laboratory solvents.

The first observation that can be made is that the cross-linked polymer materials do not dissolve in any of the selected solvents with a remaining solid content of >90 wt % for almost all samples. Polystyrene itself dissolves well in DCM, THF, and to a degree in DMF. The fact that these materials do not dissolve in these solvents is direct experimental evidence that our cross-linking reaction was effective. DCM, THF, and DMF resulted in the largest swelling compared to acetone, water, and methanol, which is understandable considering that they are good at dissolving polystyrene. What can also be observed is that the swelling ratio and the remaining solid fraction remain largely consistent for a given solvent when varying the cross-linking degree. When comparing the 100% cross-linked material with the long and short PS-TAAD, the 10-fold difference in *M_n_* does not make a big difference (Supporting, Figure S31). The most notable difference is that the shorter polymer swells slightly more in water than the higher-molecular-weight polymer. This is in line with expectation since the longer polystyrene chain imparts a more hydrophobic character to the material. The 25% cross-linked sample did show much more swelling in DMF compared to the other solvents or cross-linking degrees. This increased swelling ratio might be due to the reduced number of cross-links creating a looser network that can thus swell more easily, but in that case also an increased swelling ratio for the other solvents might have been expected as well. Alternatively, the delicate handling required to deal with these fragile solvent-swollen samples (with the associated difficulty of fully removing excess solvent) could account for the high swelling ratio.

When looking at the thermal properties of the materials, differential scanning calorimetry (DSC) revealed that the boronate-TAAD materials have a *T*_g_ of approximately 83 °C, which is slightly lower than the *T*_g_ of a polystyrene homopolymer of around 100 °C (Supporting Information, Figure S23).^64^ TGA showed that the materials follow a three-step thermal decomposition (Supporting Information, Figures S24–S27). The first, smaller step is around 183 °C, followed by the second step around 335 °C and the final, large step around 435 °C. The decomposition step around 180 °C is likely due to the decomposition of the TAAD moiety (from earlier work^41,42^ (using MeOH as a solvent instead of DMF), we could infer that this step is not related to residual DMF). From the TGA data, we can conclude that these materials are stable to elevated temperatures and suitable for hot-press molding.

**Figure 3:**
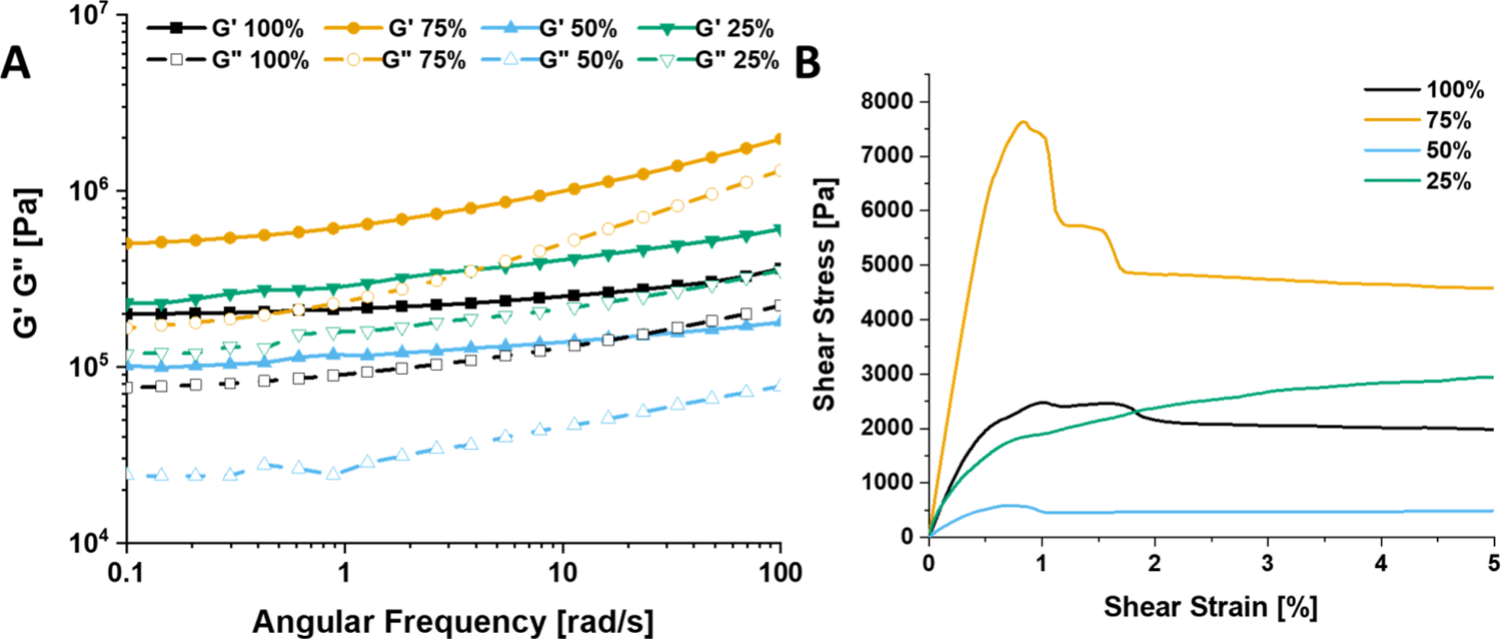
(A) Frequency sweep data of the materials with different cross-linking degrees at 100 °C with 0.1% strain from 100 to 0.1 rad/s. The solid lines with filled symbols are the storage moduli; the dashed lines with the empty symbols are the loss moduli. (B) Shear measurements of the materials with different cross-linking degrees at 100 °C. The full measurements until 10% strain can be found in Supporting Information, Figures S15–S18.

Next, the dynamic-mechanical material properties were investigated by using rheology. Frequency sweeps of the polymer networks with different cross-linking degrees, as shown in Figure 3A, showed that all four materials had a storage modulus (*G*′) around 10^5^–10^6^ Pa at 100 °C. The loss moduli (*G*″) was lower than the storage modulus for all cases, thus showing that the materials respond more like solids. No crossovers for the *G*′ and *G*″ could be observed within the measurement window. This contrasts with the frequency sweep obtained from neat polystyrene (97 kDa), which did reveal a crossover frequency (Supporting Information, Figure S37A). It was expected that the degree of cross-linking would positively correlate with the *G*′, but this does not seem to be the case. To explain this observation, we propose that a combination of various factors, including—but not necessarily limited to—the variable degree of cross-linking, a plasticizing effect of the increasing amount of PPG,^65−67^ and/or a possible phase separation between the more hydrophilic PPG polymer and the hydrophobic PS backbone, may jointly have resulted in the absent correlation between *G*′ and degree of cross-linking.

In Figure 3B, the results of the shear measurements can be seen. All samples were found to yield around 1% strain at a temperature of 100 °C. It was expected that the degree of cross-linking would correlate with an increasing shear modulus; however, again we see that the CLA acts not only as a cross-linker but also as a plasticizer. Both the cross-linking and the plasticizer have a significant impact on the shearing behavior, be it with different contributions.

**Figure 4:**
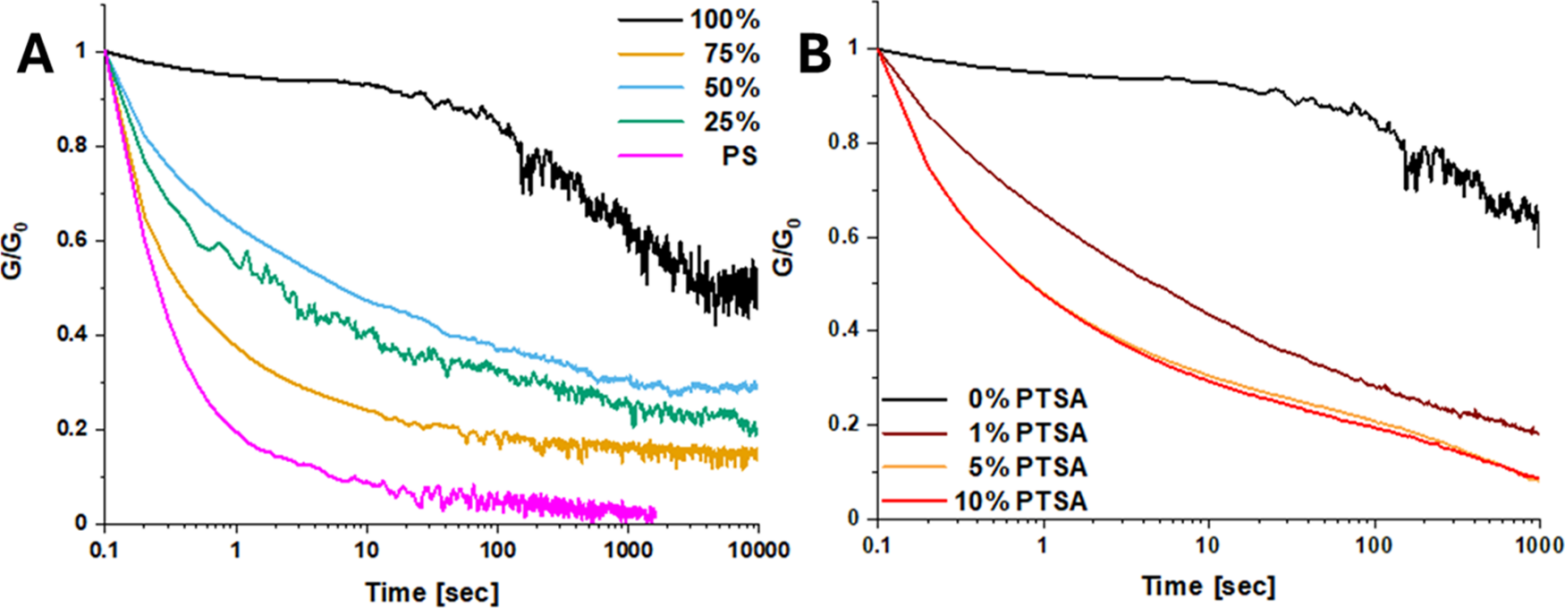
(A) Relaxation measurements of the materials with the different cross-linking degrees at 120 °C with a strain of 1%. (B) Relaxation measurements at 120 °C with 1% strain for a 100% cross-linked material with different wt % of para-toluenesulfonic acid mixed in.

The relaxation behavior of the materials at 120 °C was also studied (Figure 4A). The most notable result is the fact that these covalently cross-linked materials are able to relax stress, thus showing that they did indeed form covalent adaptable networks, since regular covalently cross-linked networks are not able to efficiently relax stress. Looking at the relaxation data of these materials, a trend can be seen where higher degrees of cross-linking correspond with longer relaxation. A notable exception can be seen in the 75% cross-linked material, which relaxes significantly faster compared to the other cross-linking degrees. Unmodified polystyrene (*M_n_* 97 kDa) was added as a reference and has the fastest relaxation of this series, as was expected since it is a similar polymer but without any cross-linking.

When comparing the relaxation time of the 100% cross-linked materials with the long and the short PS-TAAD (Supporting, Figure S33), the short polymer had a longer relaxation time. This is likely due to the short polystyrene chain being less flexible than the longer chain and thus showing a slower relaxation.

Since boronate esters are sensitive to pH, we also studied the response of different amounts of acid mixed into the material. For this, we chose *para*-toluenesulfonic acid (PTSA). Figure 4B shows the relaxation behavior of the 100% cross-linked materials with different wt % of PTSA mixed in ranging from 0 to 10%. It was hypothesized that additional acid would decrease the relaxation time of the material either directly through catalysis of the exchange reaction or indirectly by shifting the equilibrium more toward free hydroxyl groups, which could then increase the exchange rate. This is also what we observed, as the relaxation time got shorter with increasing PTSA content. Increasing the PTSA wt % from 5 to 10 wt % did not result in faster relaxation, suggesting a limit to the effect PTSA has on the network relaxation. This effect was also shown to work in materials prepared by using the short PS-TAAD polymer, where the relaxation time decreased with increasing PTSA content (Supporting Information, Figure S36). This is in contrast to the observed relaxation behavior of similar networks with boronate-TAAD linkages prepared from small linkers we studied previously.^42^ In those networks, no simple trend was observed concerning the amount of acid added and the relaxation behavior of the material. As the materials in this study contain largely the same functional groups it supports the hypothesis that matrix effects might have played a role in the effectiveness of the mixed-in acid, as was also put forward in our earlier work.^42^ Unfortunately, the fragility of the polystyrene backbone in combination with the bulky, polar TAAD side groups disrupted the temperature dependency of the network stress relaxation and thus prevented the construction of an Arrhenius plot (even with acid mixed in), thus no activation energy could be determined for these materials.^68^

**Figure 5:**
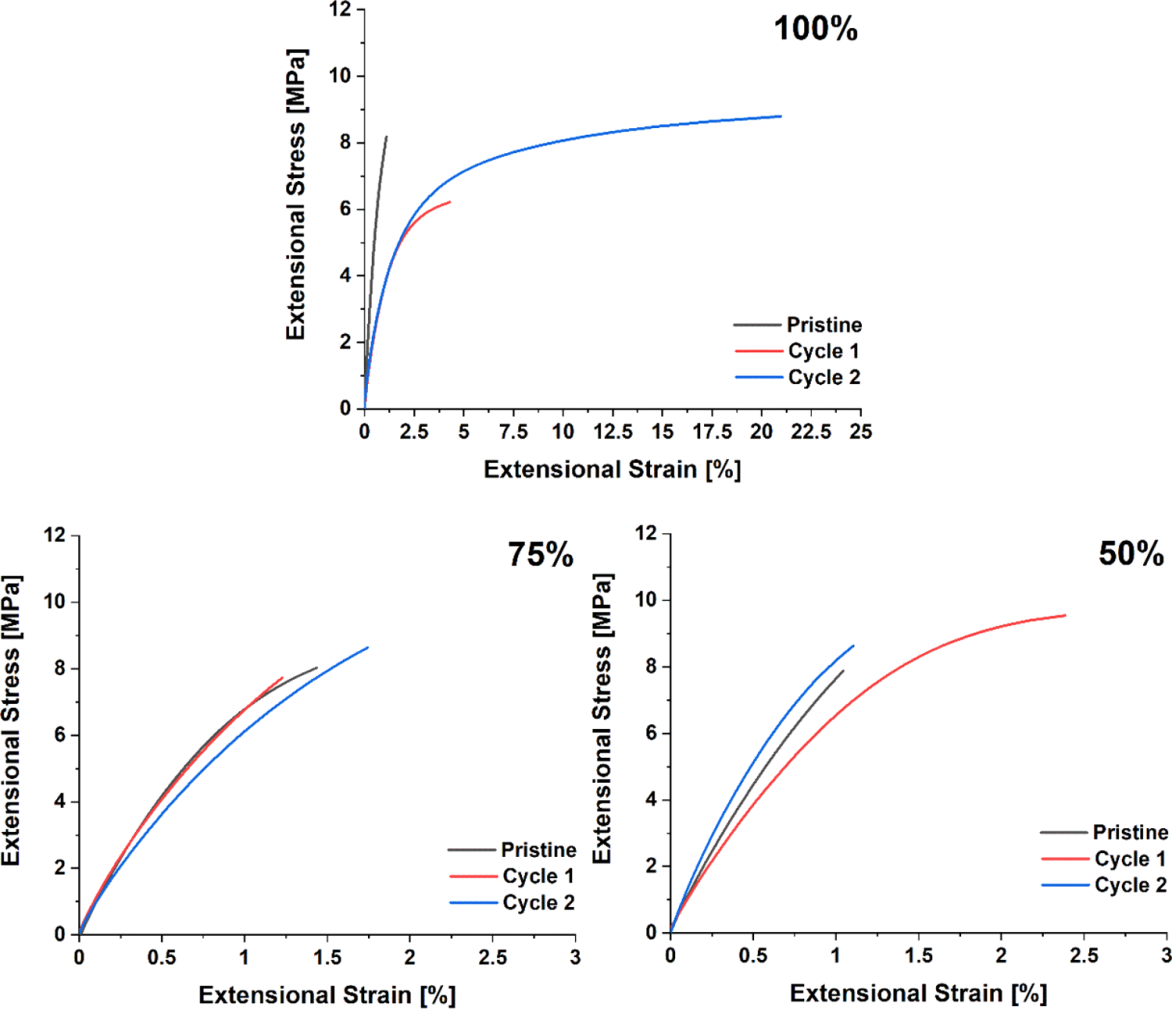
Extensional DMA measurements on the pristine and recycled materials with different cross-linking degrees. The materials with 25% cross-linking were too brittle to be measured and as such are not included. After breakage, the materials were reprocessed by heating the materials in a hot-press at 145 °C for 2 h at 5 ton pressure in a Teflon mold.

Lastly, the reprocessability of the materials was tested using extensional DMA at room temperature. The samples were cut in half and reprocessed in a Teflon hot-press mold (20 × 5 × 1 mm^3^) for 2 h at 145 °C and 5 ton. The samples were reprocessed for two cycles, as shown in Figure 5. The samples with 25% cross-linking were too brittle at room temperature to be clamped effectively. This could be due to these samples having the least amount of plasticizer and thus retaining mostly the characteristics of a brittle cross-linked polystyrene material. The data shows that even after reprocessing, the materials behave quite similarly to the pristine material for all three tested cross-linking degrees with an average Young’s modulus over all reprocessing steps of 6.2 ± 0.1, 7.8 ± 0.1, and 8.8 ± 0.1 MPa for the 100, 75, and 50%, respectively. The 100% cross-linked material does become more extensible in the second reprocessing cycle, far more than is seen for the other cross-linking degrees; however, the maximum stress remains largely the same. This suggests that the network structure has been affected by the reprocessing steps. This could be due to some accumulative damage to the TAAD moieties at the elevated reprocessing temperature, thus resulting in a lower effective cross-linking density after repeated reprocessing cycles. Another possibility would be that the network becomes less entangled through polymer alignment during the reprocessing steps. The 100% cross-linked material with the short PS-TAAD polymer was also prepared and tested; however, their increased stiffness and slower relaxation made it difficult to efficiently reprocess these materials, although it was possible to reprocess the materials for at least one cycle (Supporting Information, Figure S39).

## Conclusions

In this study, we successfully prepared and characterized a covalently cross-linked, yet recyclable, polystyrene-based thermoset using established, controlled RAFT polymerization. To do so, we prepared a dynamic-covalently cross-linked polymer entirely composed of polystyrene in its backbone and integrated the dynamic covalent boronate-TAAD chemistry to accomplish dynamic cross-links. The material was able to relax stress with and without the use of a catalyst. The material properties could be tuned through both the degree of cross-linking and the addition of an acid catalyst. The materials were demonstrated to be reprocessed over at least two cycles using hot-pressing. We believe that this work demonstrates that dynamic covalent bonds can transform tried-and-tested commodity polymers (polystyrene in our case) into recyclable and adaptable thermosets with dynamic properties. In this regard, our approach offers a promising platform for polymer waste reduction and for enhancing the circularity of polymers while also promoting the growing field of covalent adaptable networks.

## Supporting Information Available

Experimental procedures and chemical and physical characterization data (PDF)
