# Supplementary material for: Recyclable Covalent Adaptable Polystyrene Networks Using Boronates and TetraAzaADamantanes
Source: ACS Appl Polym Mater. 2024 Jun 29;6(13):7918–25. doi: 10.1021/acsapm.4c01633 (PMC11250031; doi:10.1021/acsapm.4c01633)
Supplement: Supplementary file 1 — ap4c01633_si_001.pdf [file ap4c01633_si_001.pdf]

Supporting information for

Recyclable Covalent Adaptable Polystyrene-Networks using Boronates and TetraAzaADamantanes

Simon van Hurne, Sagar Kumar Raut, Maarten Marinus Johannes Smulders*

Laboratory of Organic Chemistry, Wageningen University, Stippeneng 4, 6708 WE Wageningen, The Netherlands

* Email: maarten.smulders@wur.nl

Contents

Chemicals S2
Equipment S2
Methods S3
NMR data S8
GPC data S12
IR data S13
Rheology and DMA data S16
DSC S23
TGA S24
Solvent study data S26
Short PS-TAAD polymer materials methods and data S30
Polystyrene reference (97 kDa) data S37
Photos reprocessing S38
References S39

Chemicals

Ammonium hydroxide ge 25% solution, chloroacetone, hydroxylamine hydrochloride and 4-vinylbenzyl chloride were bought from Fisher Scientific B.V. AIBN 0.2 M in toluene, lithium bromide (Redri-Dri), Pur-A-lyzer™ Mega 1000 Dialysis Kit, poly(propylene glycol) bis(2-aminopropyl ether) (M_n 2000), polystyrene (M_n 97 kDa), sodium borohydride (90%) and styrene (reagentplus) were bought from Merck Life Sciene N.V. 4-formylphenylboronic acid (97%) was ordered from abcr GmbH. 4-Cyano-4-[(dodecylsulfanylthiocarbonyl)sulfanyl] pentanoic acid was bought from TCI Europe N.V.

Common laboratory solvents were used from various suppliers. All chemicals were used without further purification.

Equipment

NMR

Spectra were recorded on a Avance III 400 MHz Bruker NMR (101 MHz for ^{13}C), operated at 298K.

IR

Bruker TENSOR 27 Platinum FTIR spectrometer in Attenuated Total Reflection (ATR) mode, controlled by Bruker's OPUS software. Spectra were recorded with a resolution of 4 cm^{-1} and were averaged over 16 scans. The scan range was set to 4000–400 cm^{-1}.

Rheology & DMA

Rheology experiments were done on an Anton Paar 702e space rheometers using 10 mm parallel plate geometries using a N_2 driven piezo oven for heating.

DMA was performed on the 702e space using the linear drive setup at ambient temperature with extensional clamps.

The LVR of the samples was determined and all measurements were performed in the linear viscoelastic regime.

Hot-press

Samples were hot-pressed in a Specac Atlas Series Heated Platens with a WEST 6100+ Temperature Controller Unit in a Teflon mold.

Organic GPC

Agilent 1200 Organic GPC + RI detector (and ELSD); Column: PLgel 5 μm MIXED-D (Linear MW Operating Range (g/mol) 200 - 400,000) in THF. Flow rate was set to 1 mL/min. Time limit to 12 min. The concentration of the sample was 1 mg/mL. The injection volume was set to 25 μL. A calibration curve was prepared using polystyrene.

DSC

DSC was measured using a Perkin Elmer DSC 8000 from room temperature to 200 °C. Two heating cycles were recorded.

TGA

TGA was measured using a Perkin Elmer Simultaneous Thermal Analyzer (STA) 6000. Measurements were performed from room temperature to 900 °C.

Methods

Synthesis of TRISOXH$_3$ (as reported by Golovanov *et al.* 2018)[S1]

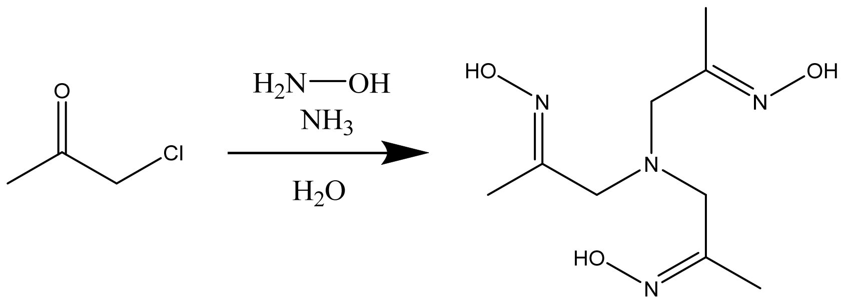

Supplementary Scheme S1. *Synthesis of TRISOXH$_3$.*

In a 250-mL round bottom flask 10.2 g (146.8 mmol) of hydroxylamine hydrochloride was dissolved in 60 mL water. 60 mL of a 25% ammonium hydroxide solution in water was added. The flask was put in a water bath to provide passive cooling. Then 11.8 mL (146.8 mmol) chloroacetone was added via syringe. The reaction was stirred for 1 hour, after which the white precipitate was filtered off and washed with plenty of water and finally with diethyl ether. The white powder was then dried overnight at 50 °C in a vacuum oven. This resulted in a fine white powder (3.38 g; 29.7%).

^{1}H NMR (400 MHz, DMSO) δ 10.57 (s, 3H), 2.90 (s, 6H), 1.74 (s, 9H).

Synthesis of 4-vinylbenzyl bromide (as reported by Chakma *et al.* 2019)[S2]

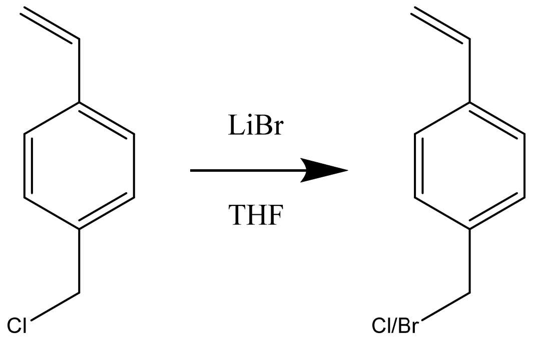

Supplementary Scheme S2. *Synthesis of 4-vinylbenzyl bromide.*

In an oven heated 100-mL round bottom flask 10 g (115.1 mmol) of lithium bromide was dissolved in 50 mL anhydrous THF. To this solution 3.7 mL (26.3 mmol) of 4-vinylbenzyl

chloride was added. This mixture was purged with N_2 for 30 min while in an ice bath. The solution was allowed to stir at room temperature for 24 hours. The solvent was evaporated and the residue was taken up in 30 mL water. The new yellow solution was extracted with 30 mL diethyl ether, which was then washed 4 times with 30 mL brine. After washing the organic layer was dried with magnesium sulfate and evaporated to yield a light-yellow oil. This oil was then resubmitted to the same procedure in the place of the 4-vinylbenzyl chloride to increase the bromide conversion. After three cycles in total, a yellow oil was obtained (2.84 g; 61.4%). The ratio bromide/chloride was determined via ^{1}H-NMR and was found to be 0.98.

^{1}H NMR (400 MHz, CDCl3) δ 7.38 (m, 4H), 6.71 (dd, J = 17.6, 10.9 Hz, 1H), 5.77 (dd, J = 17.6, 0.9 Hz, 1H), 5.29 (dd, J = 10.9, 0.9 Hz, 1H), 4.59 (s, 0H CH_2Cl), 4.50 (s, 2H CH_2Br).

Polymerization of styrene and 4-vinylbenzyl bromide

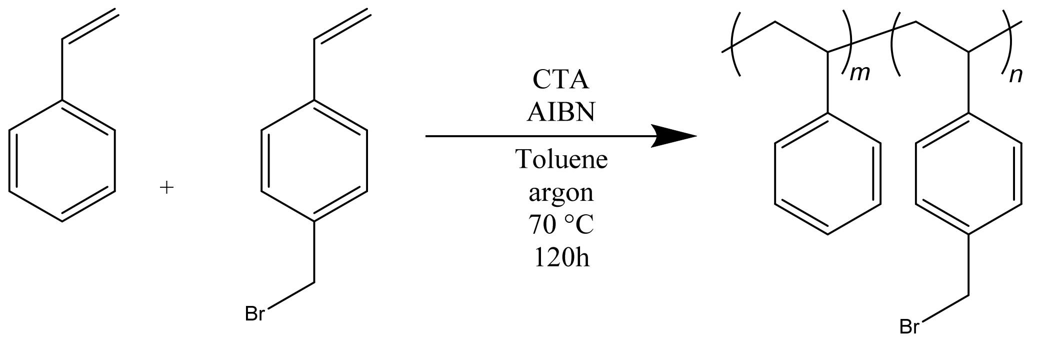

Supplementary Scheme S3. *RAFT polymerization of styrene and 4-vinylbenzyl bromide.*

10.8 mL (93.9 mmol) of styrene (after inhibitor removal via basic aluminum oxide column) and 1.0 g (5.2 mmol) of 4-vinylbenzyl bromide were dissolved in 5 mL anhydrous toluene in a 50-mL round bottom flask. 0.020 mL (0.0033 mmol) of 0.2 M AIBN in toluene and 7.2 mg (0.0198 mmol) of 4-cyano-4-[(dodecylsulfanylthiocarbonyl)sulfanyl]pentanoic acid were added. The flask was then sealed with a septum and parafilm. The mixture was then cooled in an ice bath and purged with N_2 for 30 minutes. After purging, an argon balloon was added and the reaction mixture was heated at 70 °C for 120 hours. The reaction was quenched by introduction of oxygen and the viscous liquid was diluted with 5 mL THF. The polymer was then precipitated into ice cold methanol 2 times and finally dialyzed against DCM overnight (cut-off 1 kDa). After solvent evaporation the polymer was dried in a vacuum oven overnight at 50 °C, resulting in 4.7 g product.

^{1}H NMR (400 MHz, $CDCl_3$) δ 7.66 – 6.20 (75H), 4.42 (s, 2H), 2.10-1.60 (13H), 1.43 (s, 29H).

Supplementary Table S1. *Characterization of polystyrene-co-4-vinylbenzyl bromide. Note that the used 4-vinylbenzyl bromide still contained 3.5% 4-vinylbenzyl chloride. The reported monomers incorporated are for both the bromide and the chloride monomer combined.*

M_n (g/mol)	86200
M_w (g/mol)	128200
Dispersity	1.5
Chain length	791
4-vinylbenzyl bromide/chloride in chain%	5.3%
4-vinyl benzyl/chain	42

TAAD functionalization of polystyrene-co-4-vinylbenzyl bromide via nitrogen quaternization (based on Golovanov *et al.*)[S1]

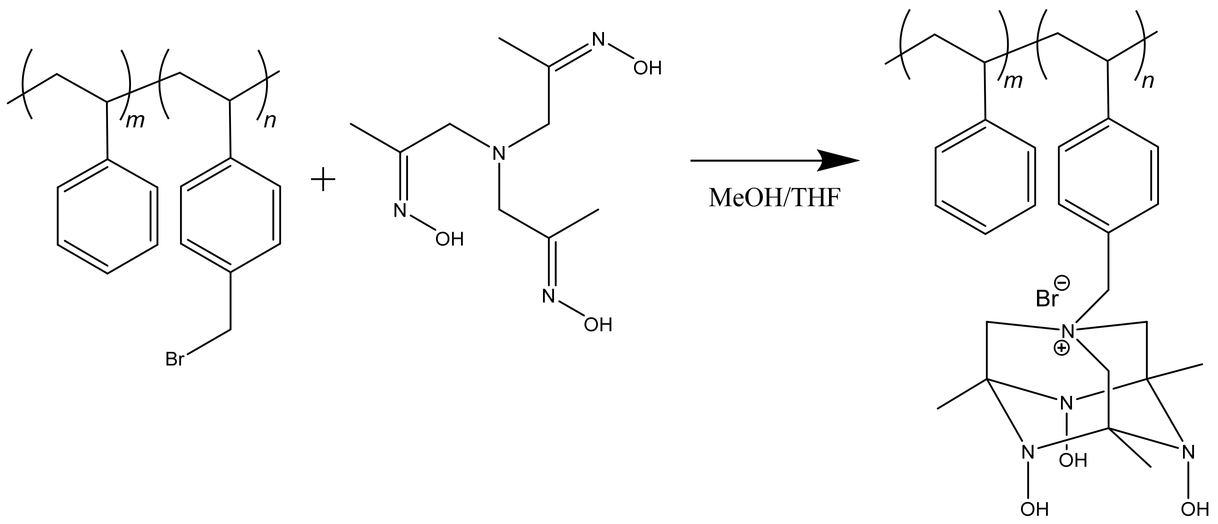

Supplementary Scheme S4. *Functionalization of polystyrene-co-4-vinylbenzyl bromide with TRISOXH$_3$ via nitrogen quaternization to produce a TAAD-functionalized polystyrene named PS-TAAD.*

4.5 g (0.052 mmol) of polystyrene-co-4-vinylbenzyl bromide and 0.76 g (3.3 mmol) of TRISOXH$_3$ were dissolved in 115 mL THF/MeOH 60/40. The reaction was then stirred for 7 days at room temperature. The volume was reduced and the product was precipitated in diethyl ether 2 times. The product was then collected via vacuum filtration and dried in a vacuum oven at 50 °C overnight. This resulted in a white powder (3.72 g, ~100% functionalization as determined by NMR).

^{1}H NMR (400 MHz, DMSO) δ 8.55 (m, 3H), 6-7.5 (74H), 4.46 (s, 2H), 2.12 – 0.71 (49H).

Synthesis of bisboronic acid crosslinker CLA (adapted from Boa *et al.* 2018)[S3]

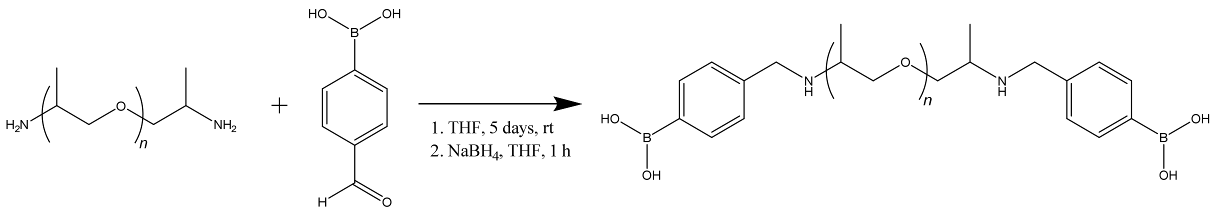

Supplementary Scheme S5. *Synthesis of boronic acid linker CLA.*

13.0 mL (6.5 mmol) poly(propylene glycol) bis(2-aminopropyl ether) M_n 2000 and 3.76 g (16.3 mmol) 4-formylphenylboronic acid were dissolved in 40 mL anhydrous THF under nitrogen and stirred for 5 days. The solution was then cooled in an ice batch. Then 0.81 g (21.4 mmol) $NaBH_4$ was added slowly. The solution was stirred for 1 hour at room temperature, after which it was dialysed (1 kDA cut-off) against H_2O/MeOH (50/50) for 2 days. After evaporation of the solvent a yellow/greenish sticky plaque was obtained (14.4 g; 86.4%)

^{1}H NMR (400 MHz, MeOD) δ 7.47 (d, J = 7.4 Hz, 4H), 7.14 (d, J = 7.7 Hz, 4H), 3.83 (dq, J = 24.3, 12.4, 11.9 Hz, 3H), 3.59 – 3.22 (m, 106H), 1.04 (dd, J = 6.5, 3.1 Hz, 105H).

^{13}C NMR (101 MHz, MeOD) δ 208.93, 163.45, 133.51, 133.18, 132.93, 132.45, 129.58, 128.21, 126.92, 126.60, 125.43, 75.54, 75.50, 75.42, 75.40, 75.31, 75.28, 75.17, 75.15, 75.08, 75.03, 74.98, 74.95, 73.30, 72.99, 72.93, 72.72, 72.69, 72.62, 72.57, 66.52, 64.27, 56.29, 56.08, 55.86, 52.30, 52.03, 51.89, 50.05, 49.96, 49.85, 48.49, 48.28, 48.06, 47.85, 47.64, 47.42, 47.21, 47.00, 46.44, 39.10, 35.57, 30.29, 28.78, 28.58, 28.39, 17.38, 17.16, 17.02, 16.35, 16.30, 16.25, 16.01, 15.84, 15.77, 14.53.

Material preparation

General protocol

100 mg (0.001 mmol polymer; 0.044 mmol TAAD) PS-TAAD was dissolved in 0.2 mL DMF. Then 49.9/37.4/24.9/12.5 mg (0.022/0.017/0.011/0.006 mmol; respectively for 100%/75%/50%/25%) CLA was dissolved separately in 0.1 mL methanol. The solutions were mixed and vortexed for 10 seconds, after which it was quickly transferred divided over 2 silicon molds (r = 10 mm, h = 1 mm) via syringe.

Note: after mixing the stock solutions, crosslinking will immediately start. Make sure you have the vortex, syringe and mold at hand when you begin. After 20-30 seconds it will be difficult for the material to exit the syringe. The solutions were mixed and vortexed for 10 seconds, after which it was quickly transferred to a silicon mold (r = 10 mm, h = 1 mm) via syringe.

Protocol for PTSA containing samples

50 mg (0.0005 mmol polymer; 0.022 mmol TAAD) PS-TAAD was dissolved in 0.1 mL DMF. Then 24.9 mg (0.011) CLA was dissolved separately in 0.1 mL methanol. Also 0.8/3.9/8.3 mg (for 1%, 5% and 10%) PTSA was dissolved separately in 0.1 mL methanol.

Solvent study

The solvent study was performed by putting ~5 mg material in 10 mL solvent and letting it swell overnight. The material was then carefully removed from the solvent, excess solvent was removed with a tissue. Then the material was placed on a weighted glass Petri dish and the swollen weight was measured. The material was then dried overnight in a vacuum oven at 50 °C after which the material was weighted again to determine the remaining fraction.

NMR data

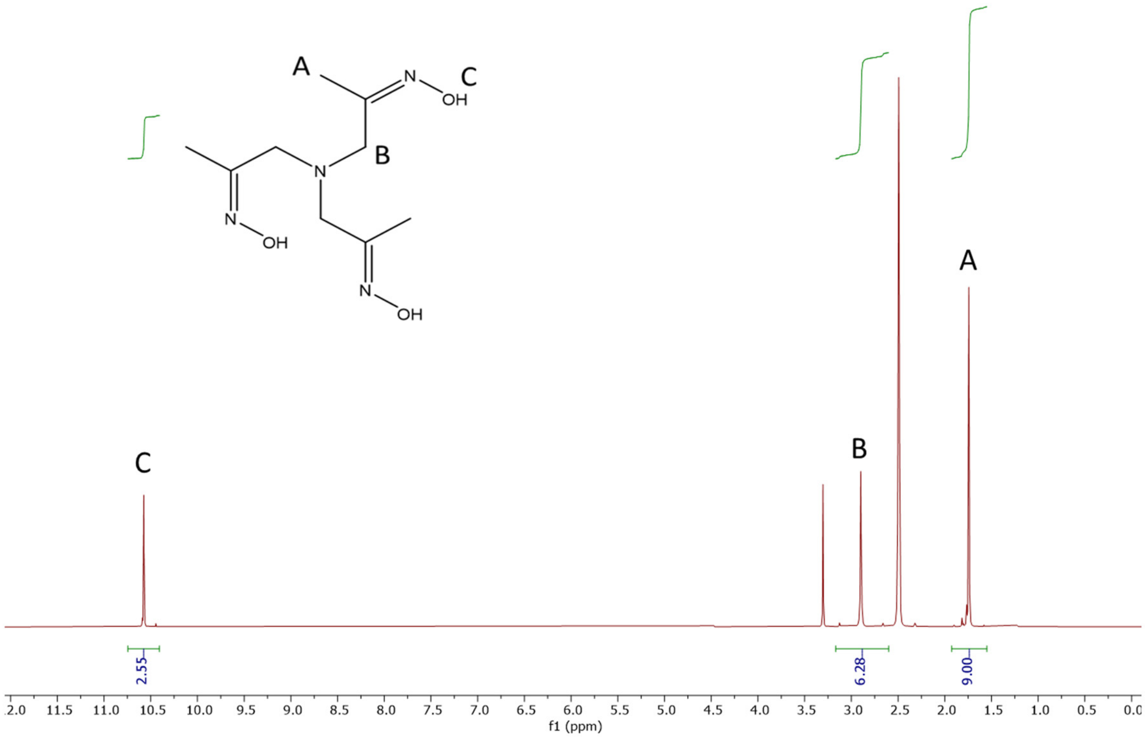

Supplementary Figure S1. *^{1}H-NMR spectrum of TRISOXH$_3$ in d$_6$-DMSO.*

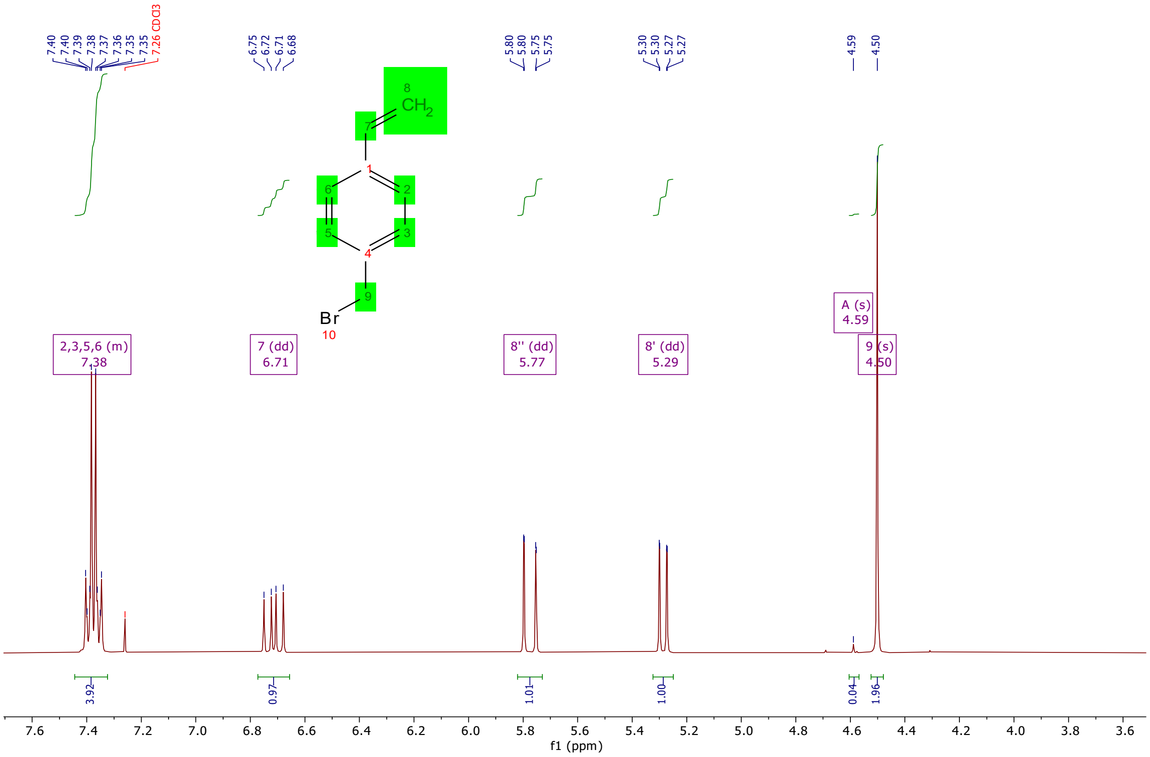

Supplementary Figure S2. *^{1}H-NMR spectrum of 4-vinyl benzyl bromide in CDCl$_3$. The peak at 4.59 ppm is coming from CH$_2$—Cl; the peak at 4.50 ppm from CH$_2$—Br.*

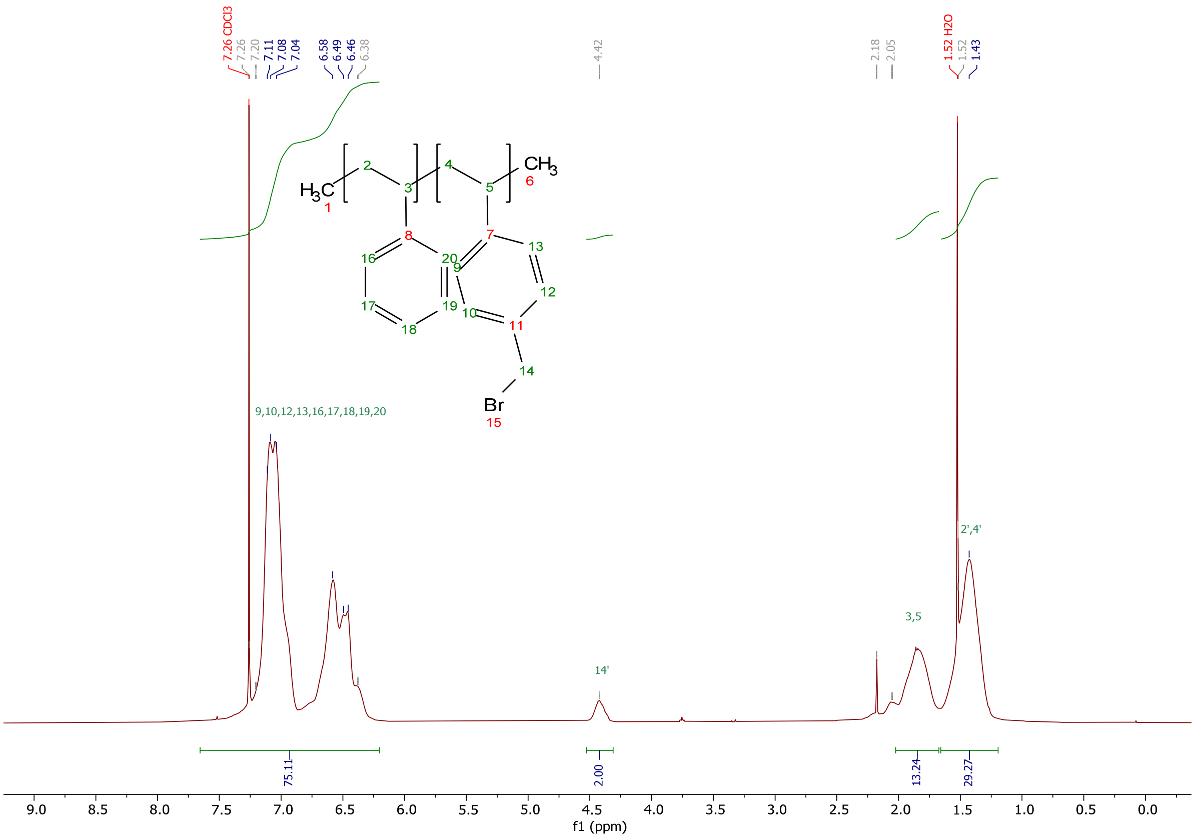

Supplementary Figure S3. *^{1}H-NMR spectrum of polystyrene-co-4-vinylbenzyl bromide in $CDCl_3$.*

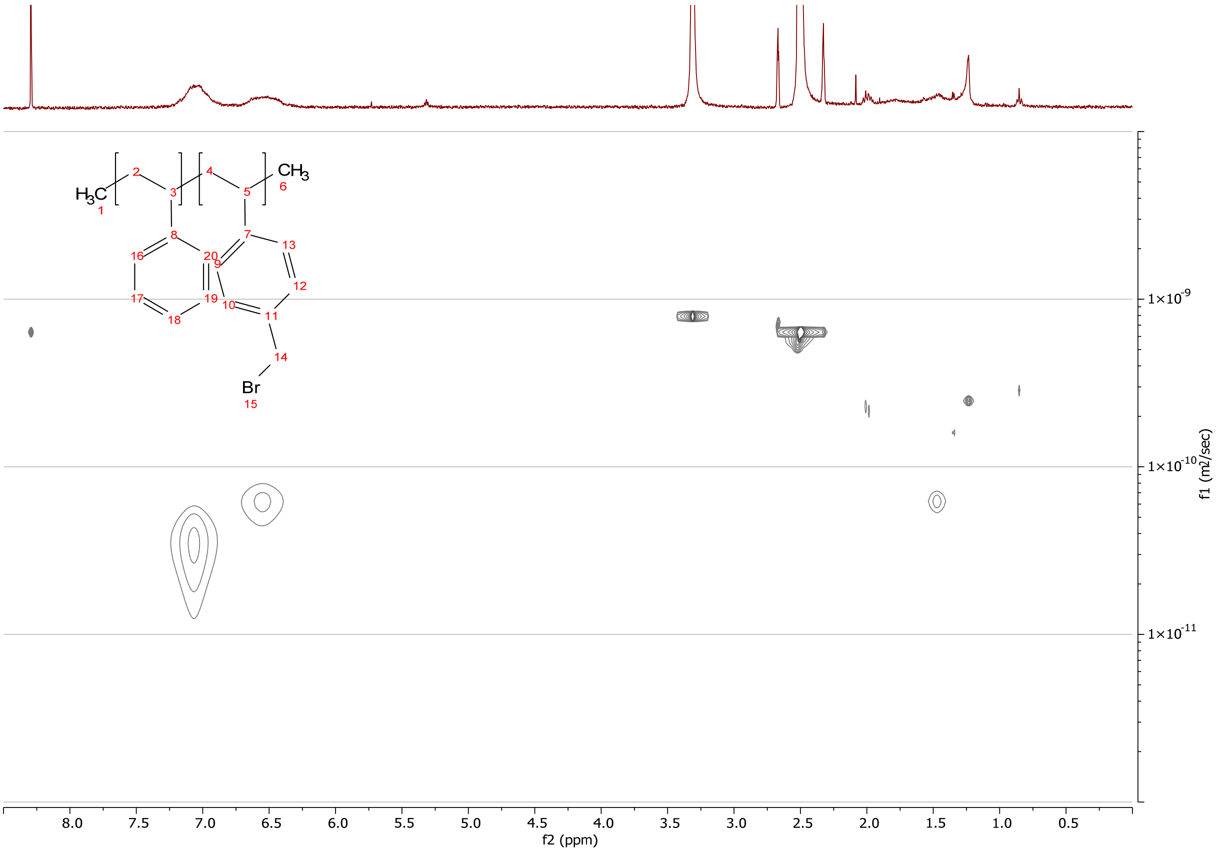

Supplementary Figure S4. *^{1}H-NMR DOSY spectrum of polystyrene-co-4-vinylbenzyl bromide in d_6-DMSO : $CDCl_3$ (9:1).*

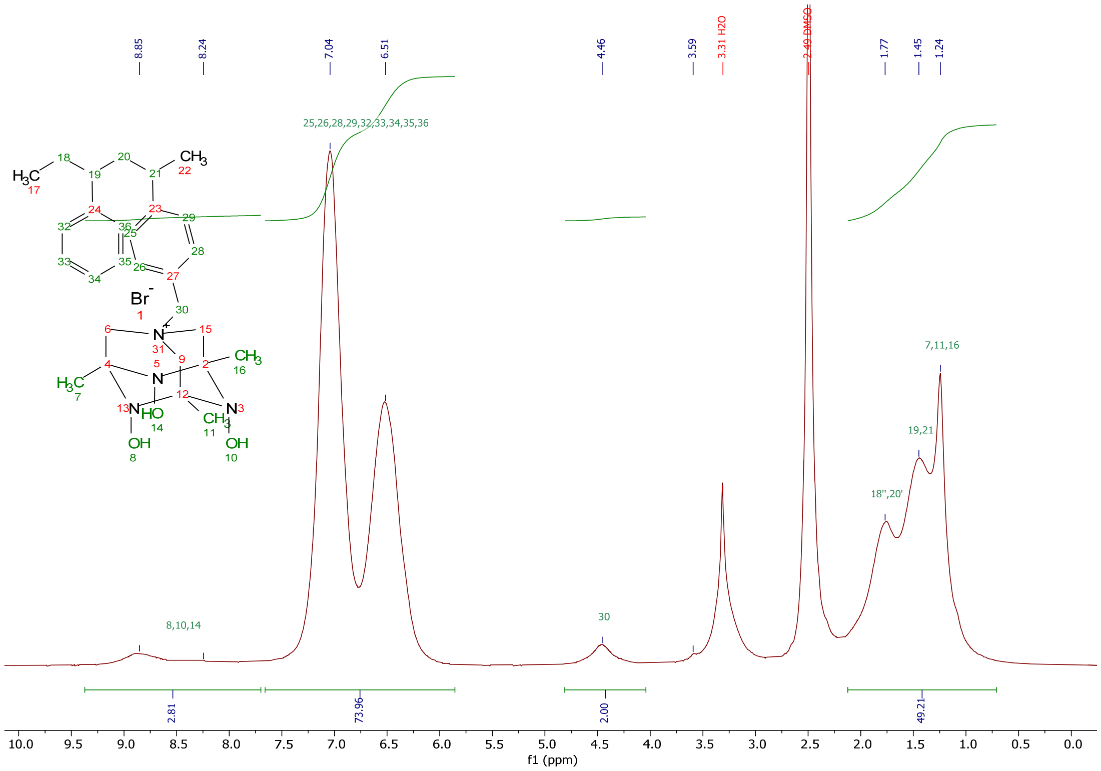

Supplementary Figure S5. *^{1}H-NMR spectrum of TAAD-functionalized polystyrene-co-4-vinylbenzyl bromide in CDCl$_3$.*

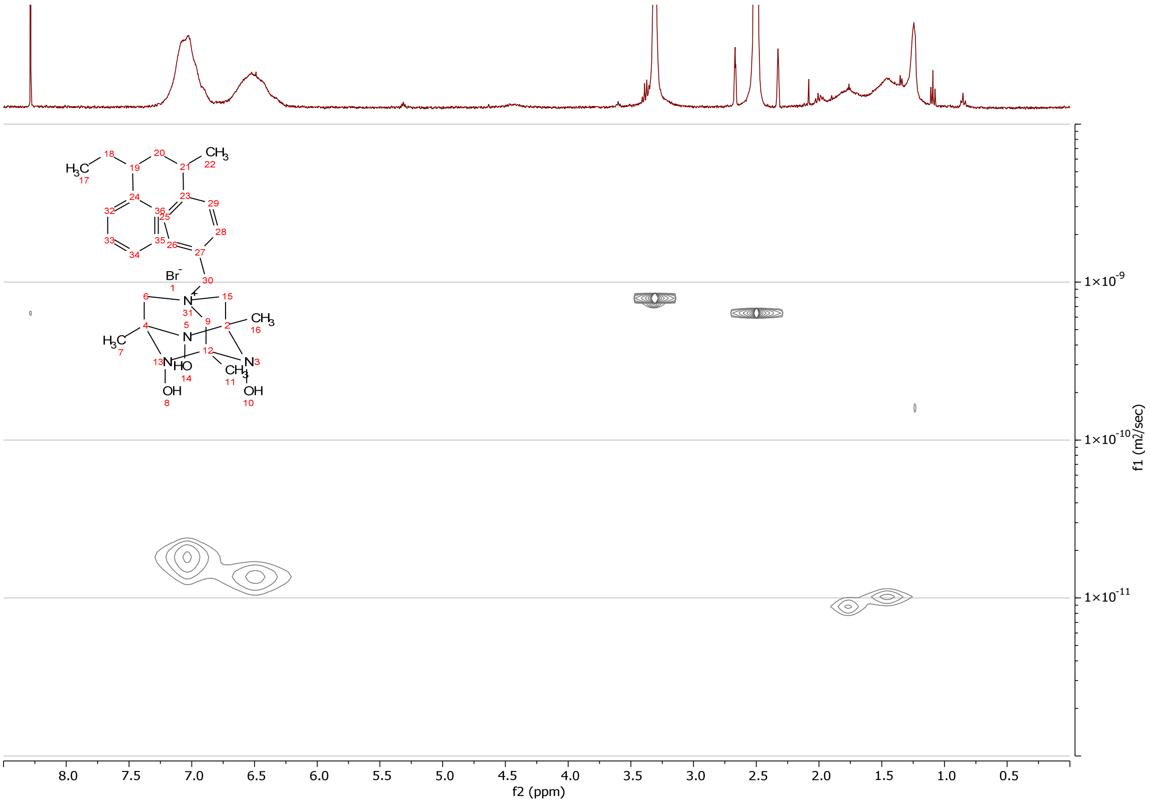

Supplementary Figure S6. *^{1}H-NMR DOSY spectrum of TAAD-functionalized polystyrene-co-4-vinylbenzyl bromide in d$_6$-DMSO : CDCl$_3$ (9:1).*

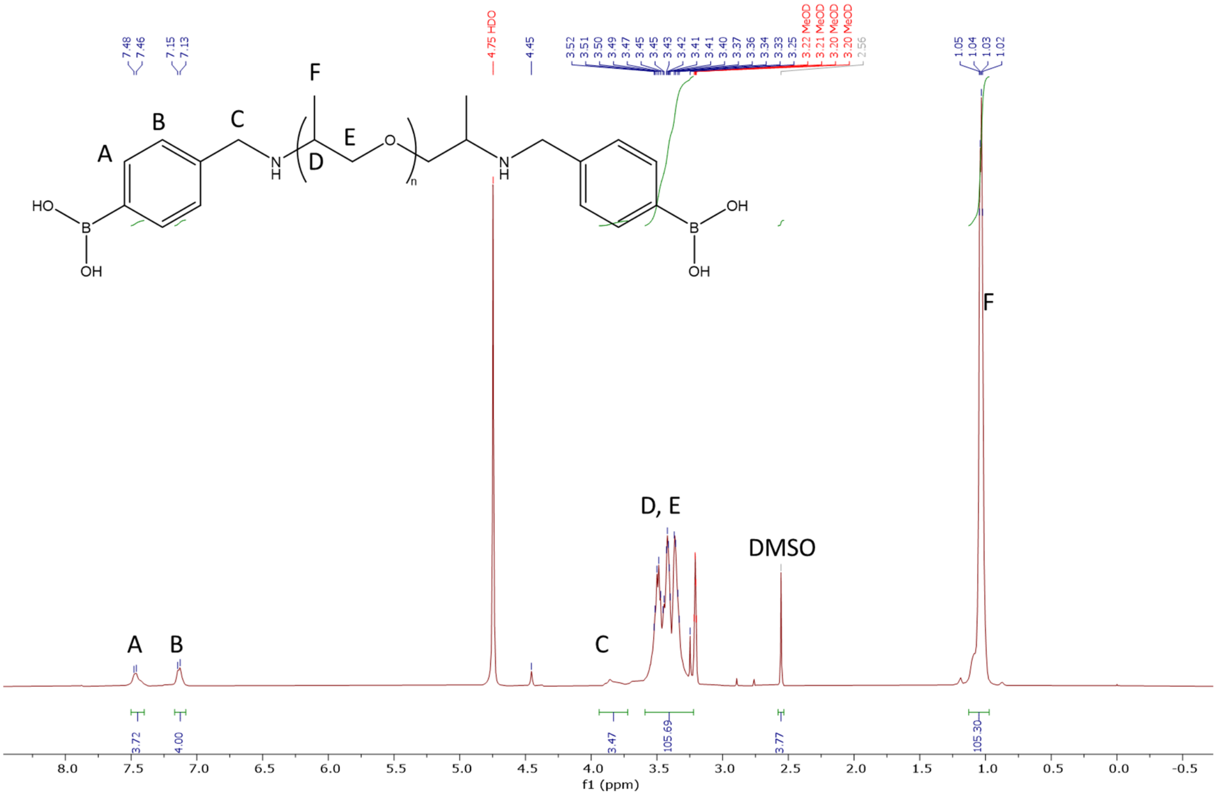

Supplementary Figure S7. *^{1}H-NMR spectrum of CLA in d$_6$-DMSO.*

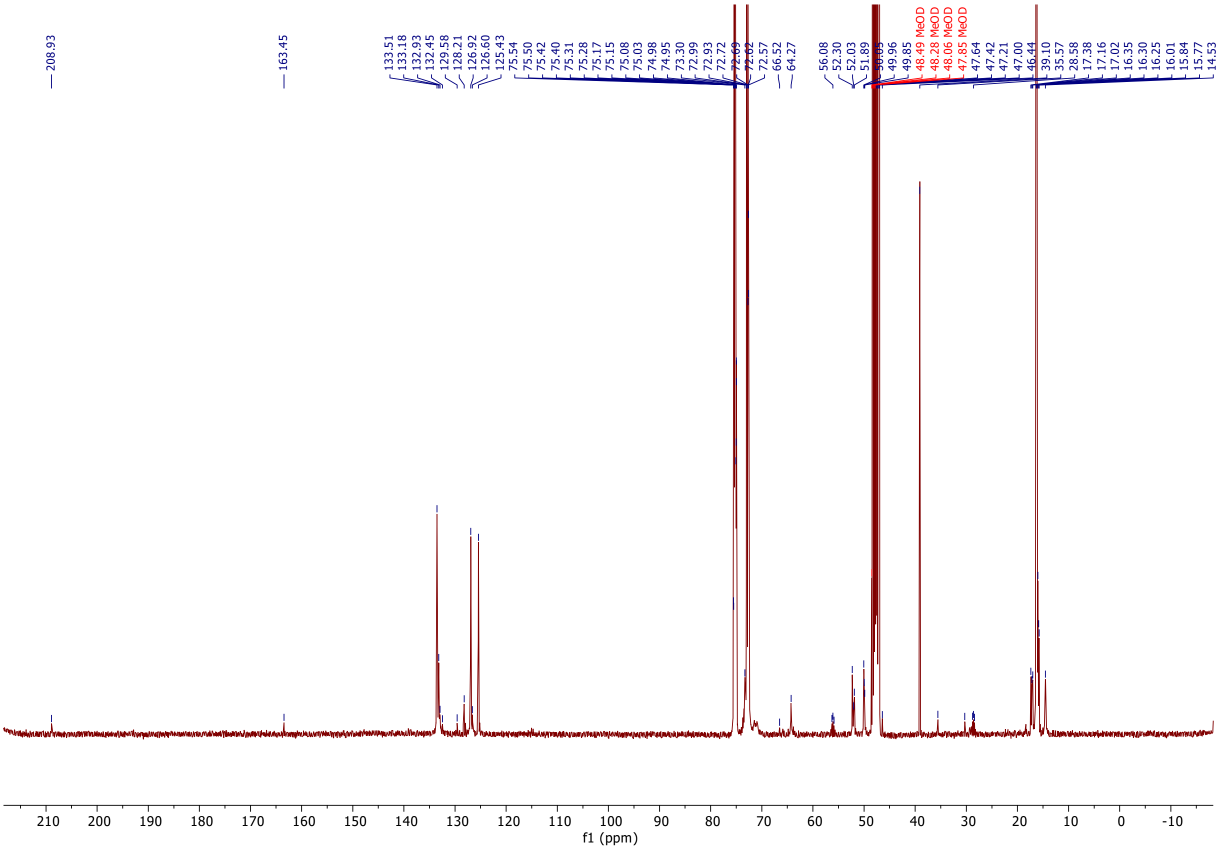

Supplementary Figure S8. *^{13}C-NMR spectrum of CLA in d$_6$-DMSO.*

GPC data

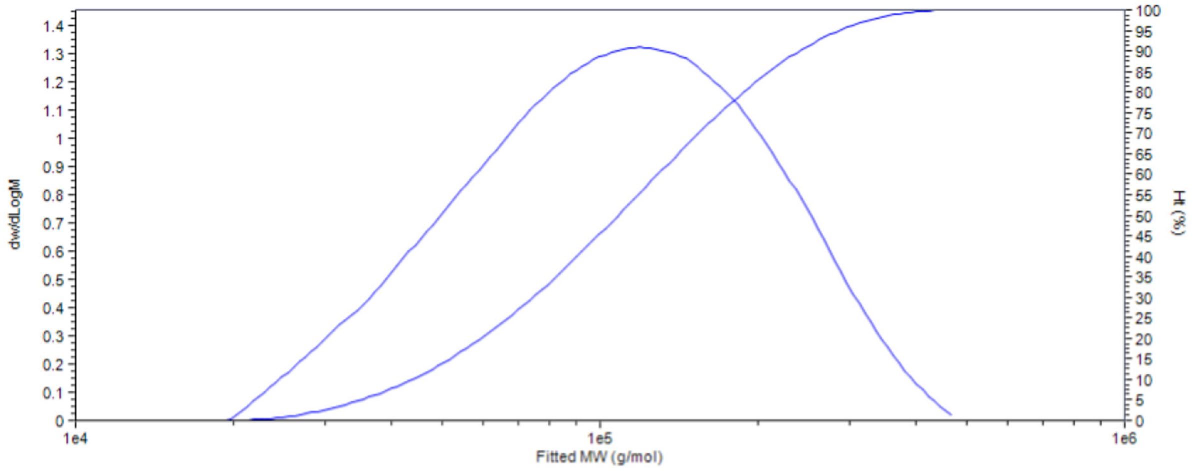

Supplementary Figure S9. *Fitted GPC data of poly(styrene-co-4-vinylbenzylbromide).*

Supplementary Table S2. *Obtained weight and dispersity for poly(styrene-co-4-vinylbenzylbromide).*

RT (min)	M_n (g/mol)	M_w (g/mol)	Dispersity
6.941	86200	128200	1.5

IR data

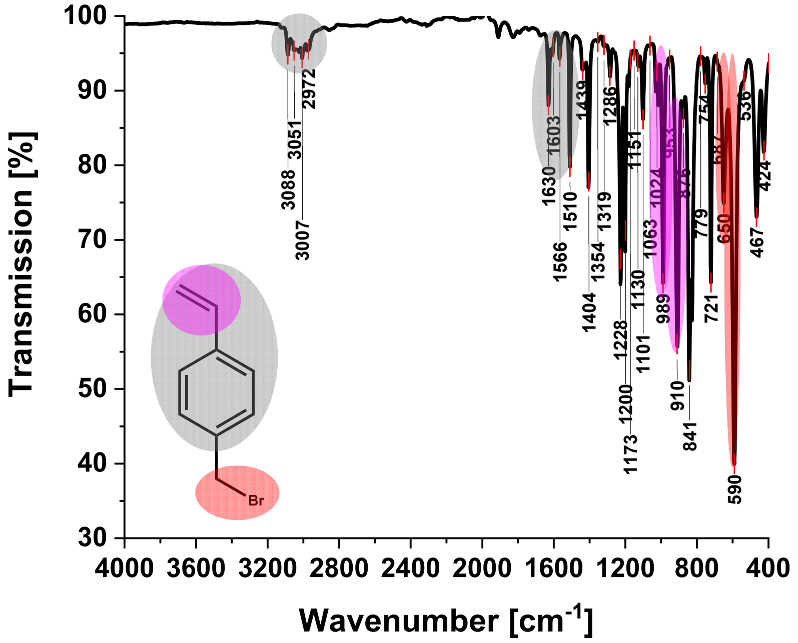

Supplementary Figure S10. *IR spectrum of 4-vinylbenzyl bromide.*

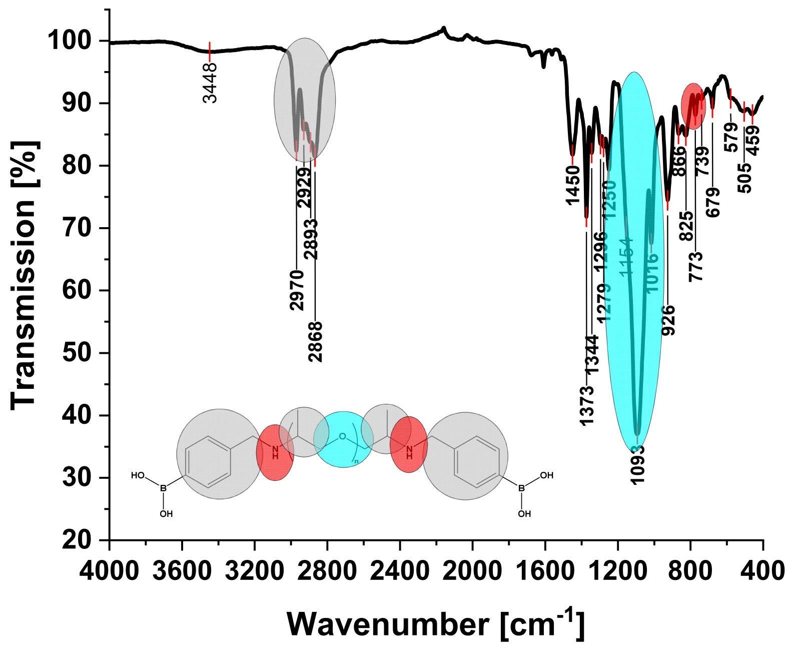

Supplementary Figure S11. *IR spectrum of CLA.*

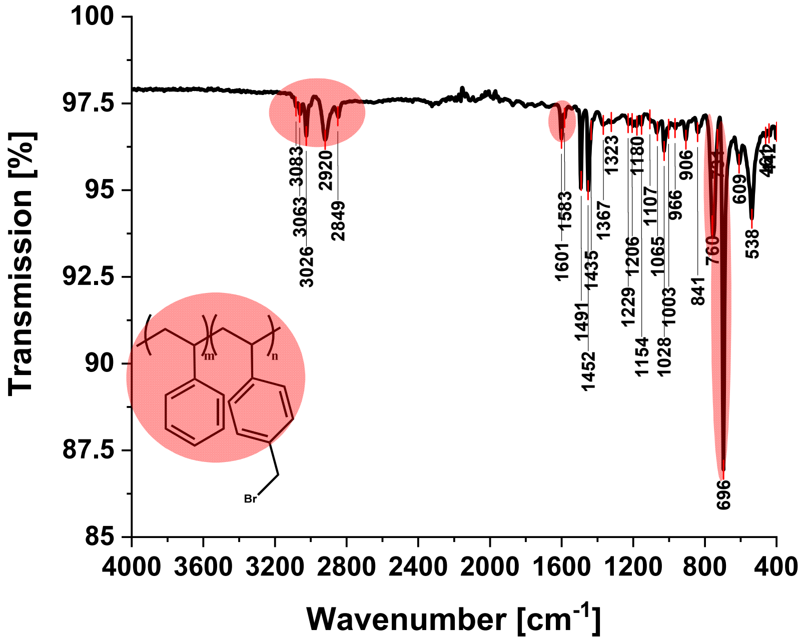

Supplementary Figure S12. *IR spectrum of polystyrene-co-4-vinylbenzyl bromide.*

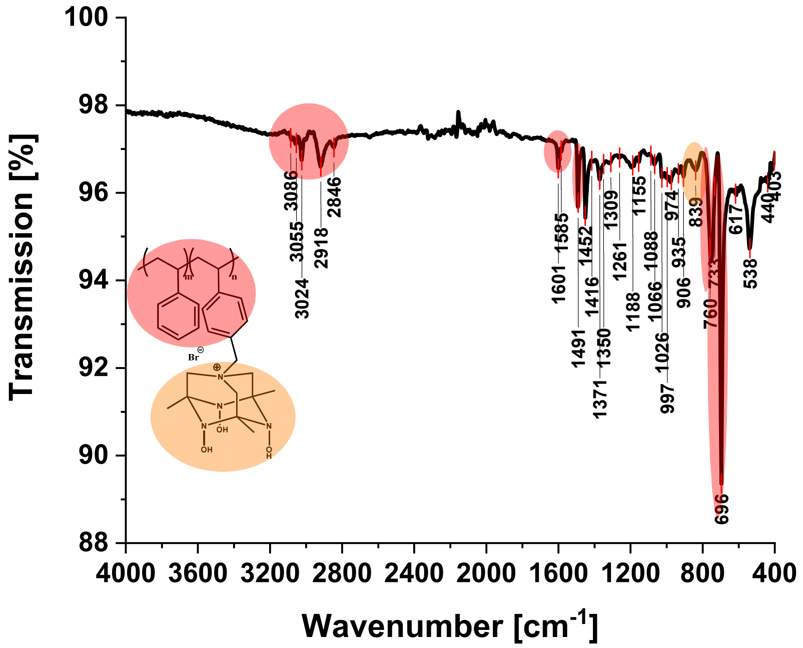

Supplementary Figure S13. *IR spectrum of TAAD functionalized polystyrene-co-4-vinylbenzyl bromide.*

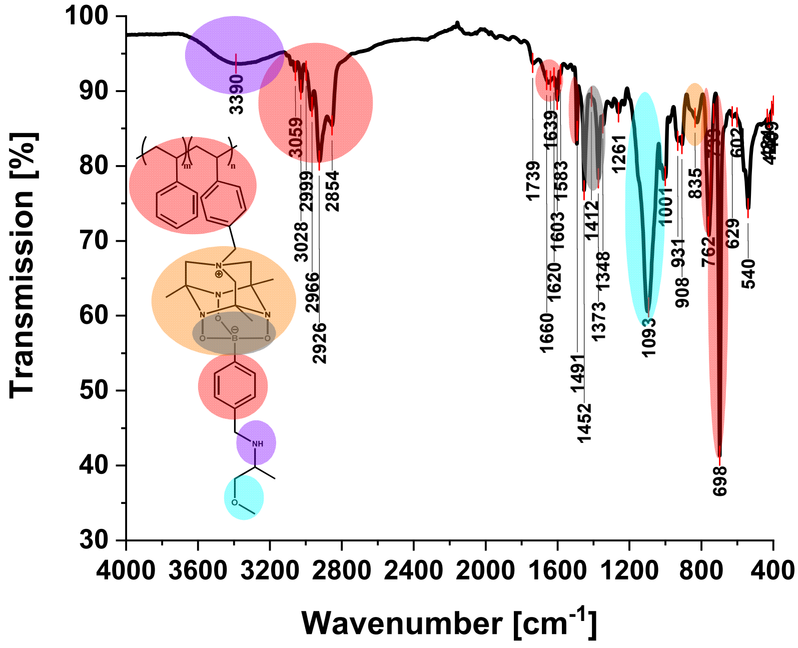

Supplementary Figure S14. *IR spectrum of a 100% crosslinked network of PS-TAAD and CLA.*

Rheology and DMA data

<u>100% crosslinked</u>

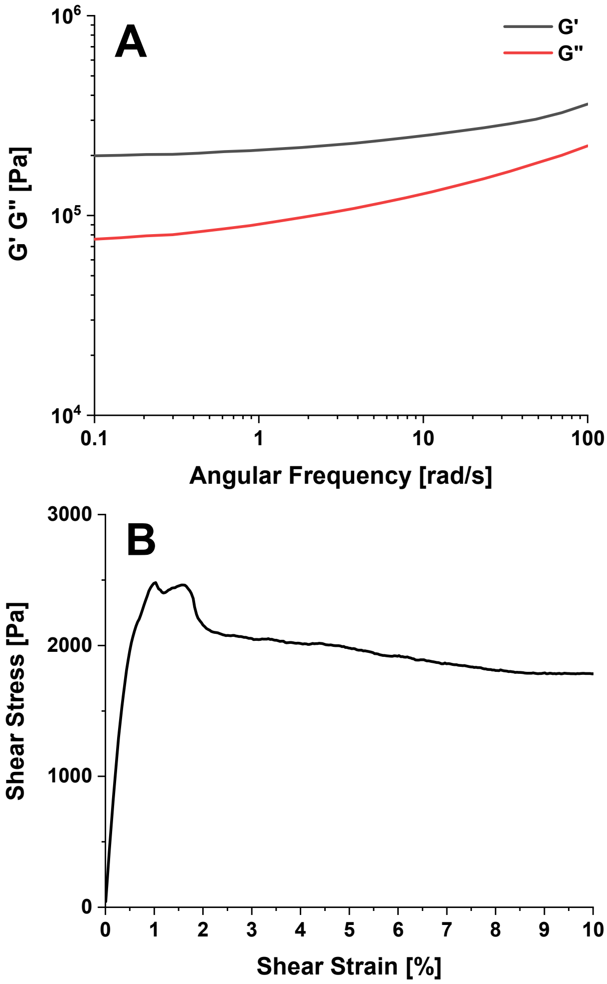

Supplementary Figure S15. *Frequency sweep of a 100% crosslinked PS-TAAD material at 100 °C with a strain of 0.1% (A). Shear experiment of a 100% crosslinked PS-TAAD material at 100 °C (B).*

75% crosslinked

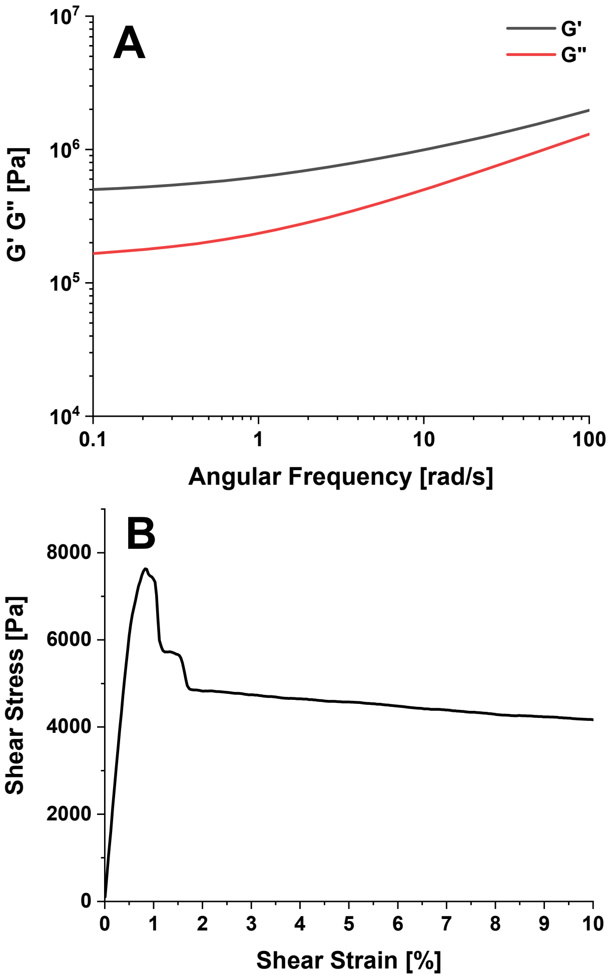

Supplementary Figure S16. *Frequency sweep of a 75% crosslinked PS-TAAD material at 100 °C with a strain of 0.1% (A). Shear experiment of a 75% crosslinked PS-TAAD material at 100 °C (B).*

50% crosslinked

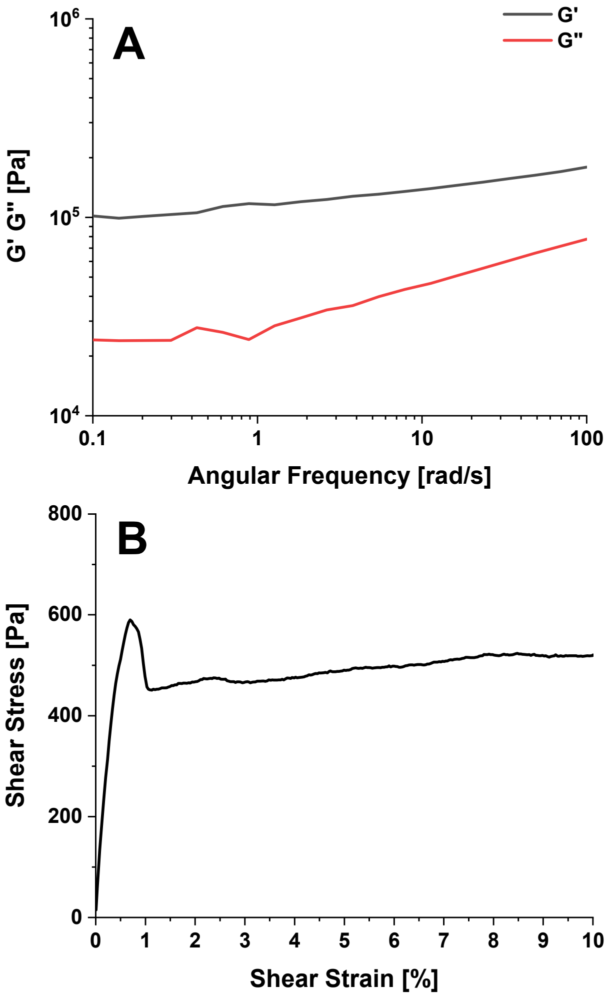

Supplementary Figure S17. *Frequency sweep of a 50% crosslinked PS-TAAD material at 100 °C with a strain of 0.1% (A). Shear experiment of a 50% crosslinked PS-TAAD material at 100 °C (B).*

25% crosslinked

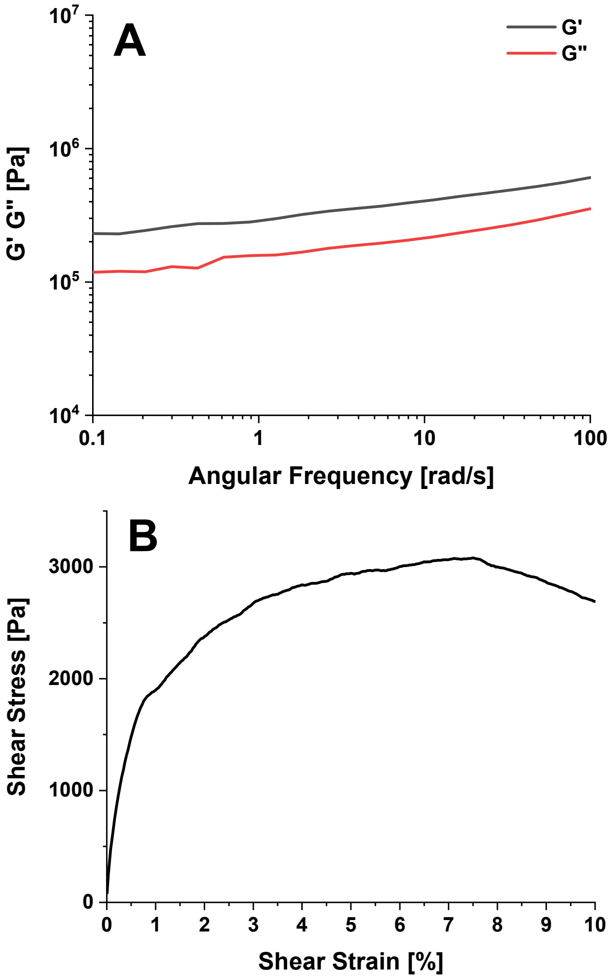

Supplementary Figure S18. *Frequency sweep of a 25% crosslinked PS-TAAD material at 100 °C with a strain of 0.1% (A). Shear experiment of a 25% crosslinked PS-TAAD material at 100 °C (B).*

<u>100% crosslinked + 1 wt% PTSA</u>

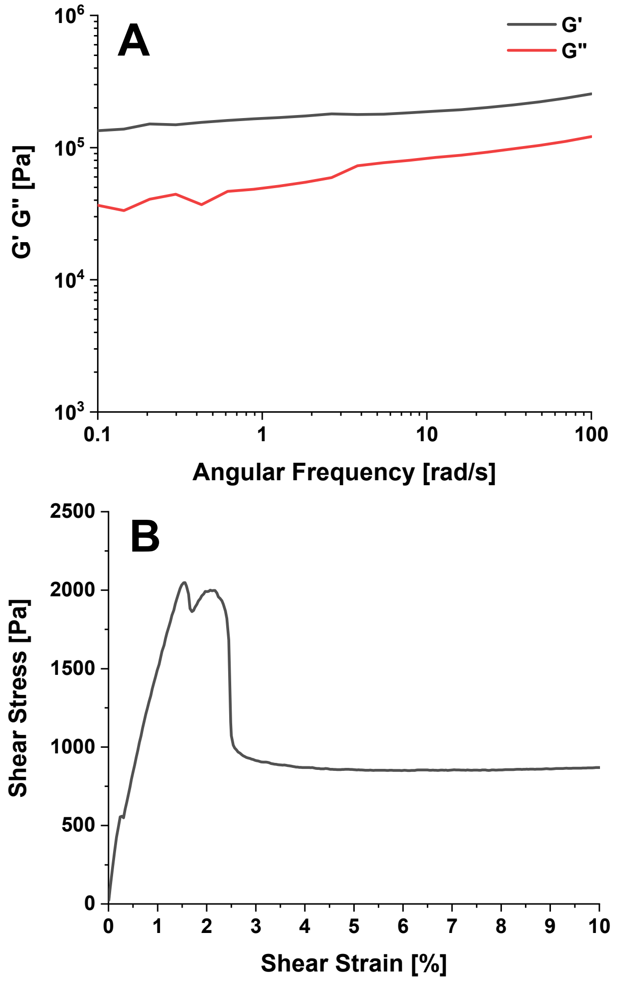

Supplementary Figure S19. *Frequency sweep of a 100% crosslinked PS-TAAD material + 1 wt% PTSA at 100 °C with a strain of 0.1% (A). Shear experiment of a 100% crosslinked PS-TAAD material + 1 wt% PTSA at 100 °C (B).*

100% crosslinked + 5 wt% PTSA

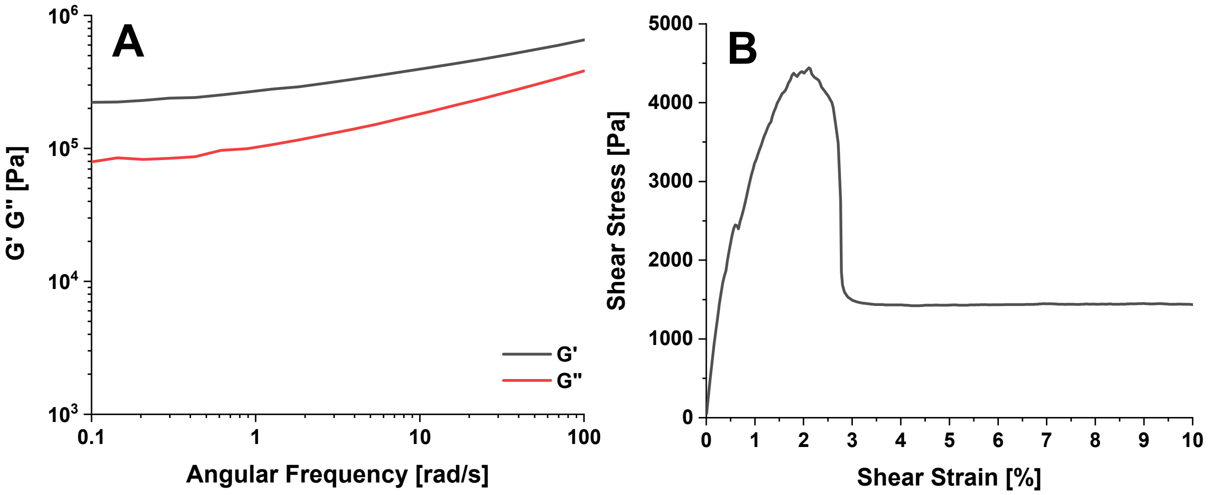

Supplementary Figure S20. *Frequency sweep of a 100% crosslinked PS-TAAD material + 5 wt% PTSA at 100 °C with a strain of 0.1% (A). Shear experiment of a 100% crosslinked PS-TAAD material + 5 wt% PTSA at 100 °C (B).*

100% crosslinked + 10 wt% PTSA

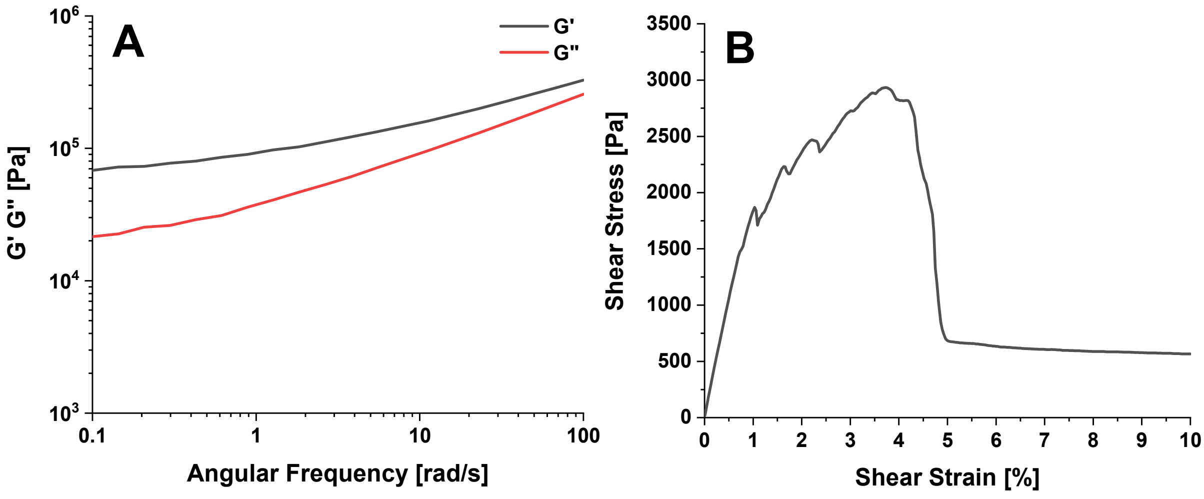

Supplementary Figure S21. *Frequency sweep of a 100% crosslinked PS-TAAD material + 10 wt% PTSA at 100 °C with a strain of 0.1% (A). Shear experiment of a 100% crosslinked PS-TAAD material + 10 wt% PTSA at 100 °C (B).*

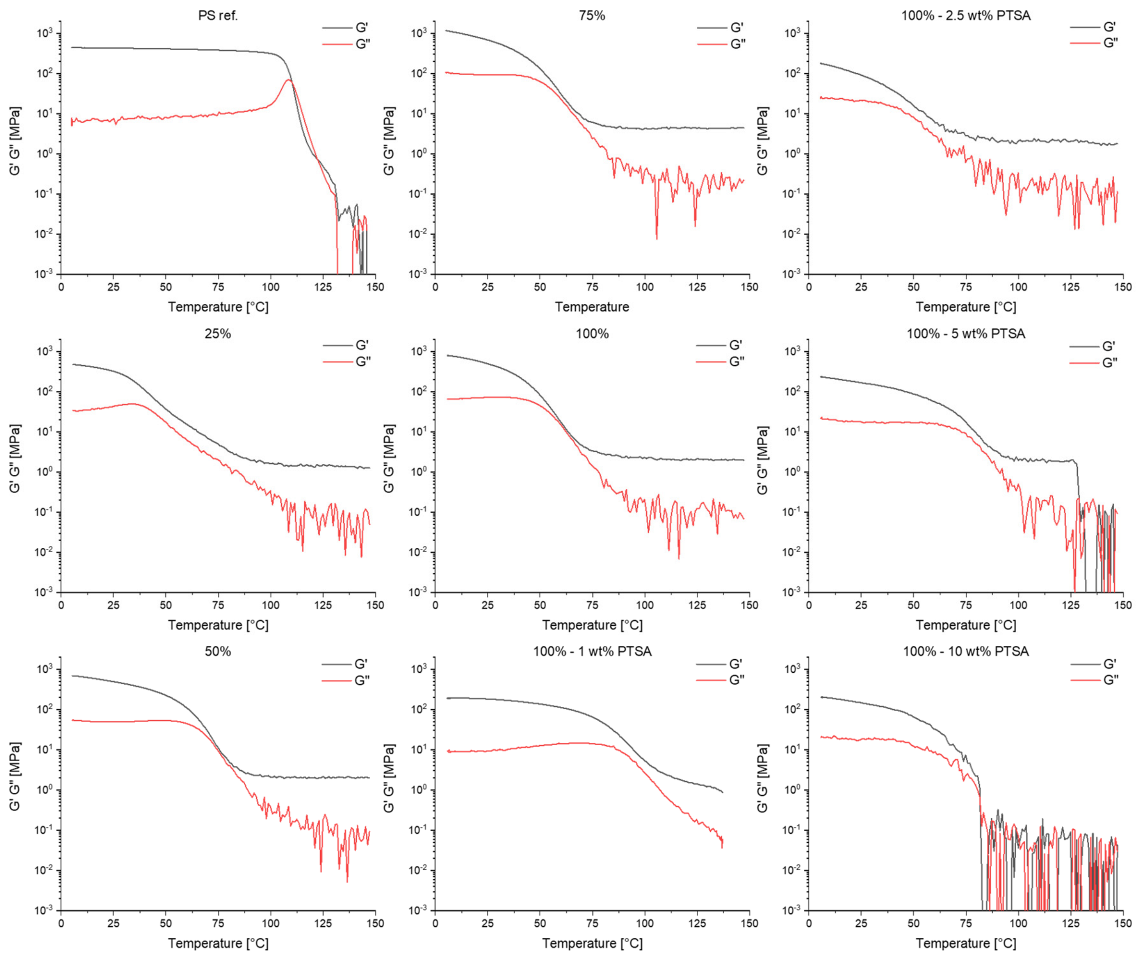

Supplementary Figure S22. *DMA temperature sweeps of the polystyrene reference, boronate-TAAD networks with varying crosslinking degrees and the networks with varying amount of PTSA mixed in. The samples were measured from 5 to 150 °C at 2 °C/min, 1 Hz and a preload force of 0.5 N.*

DSC

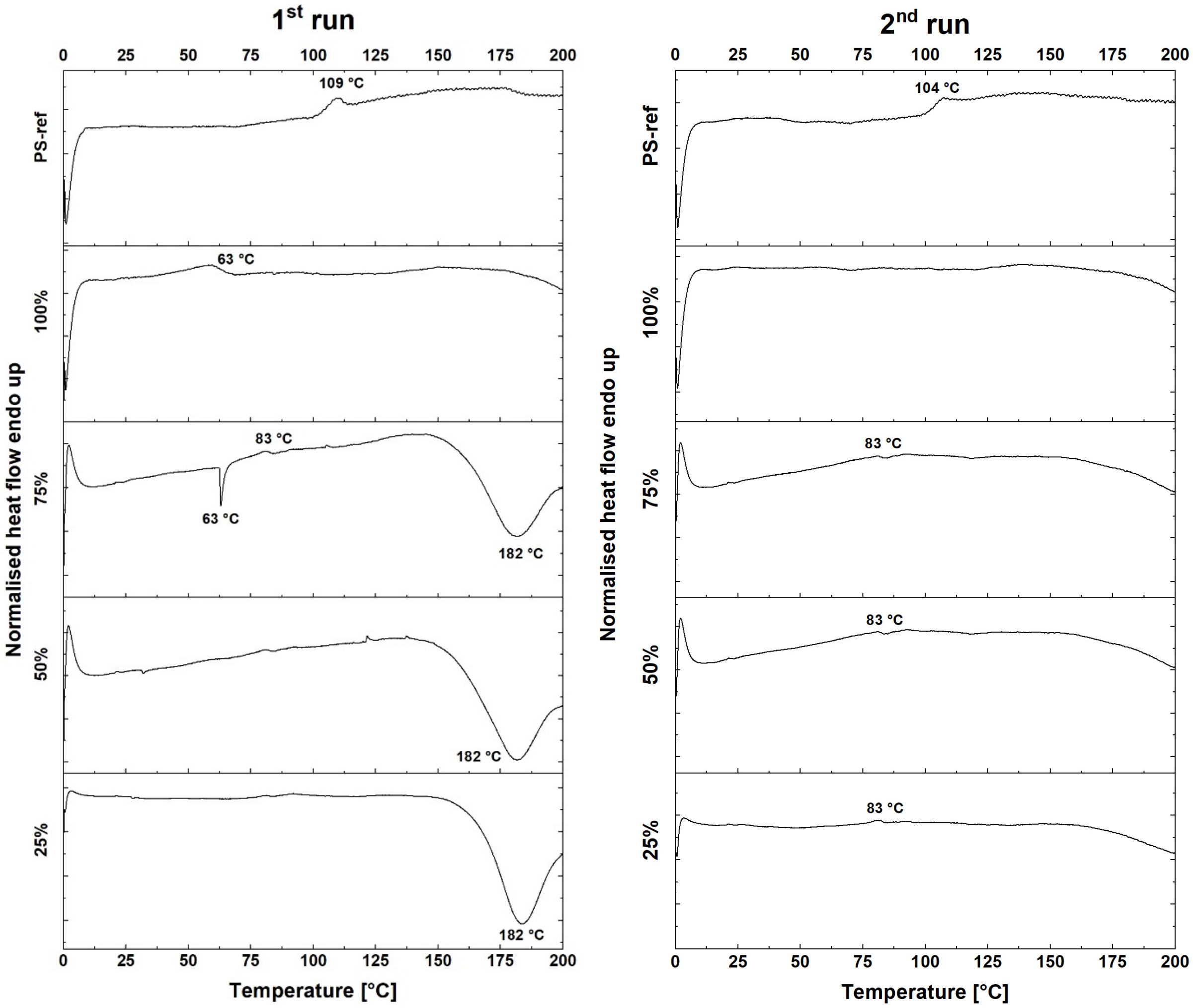

Supplementary Figure S23. *First and second DSC heating run of the polystyrene reference and the boronate-TAAD networks with the varying crosslink degrees.*

TGA

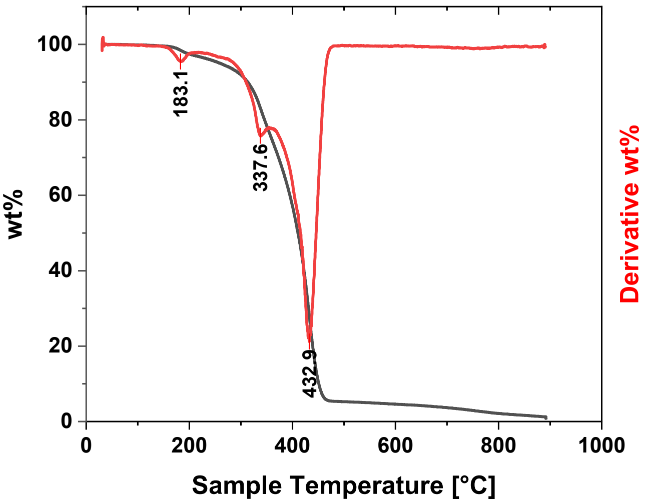

Supplementary Figure S24. *TGA measurement from room temperature till 900 °C for a 100% PS-TAAD material under* N_2.

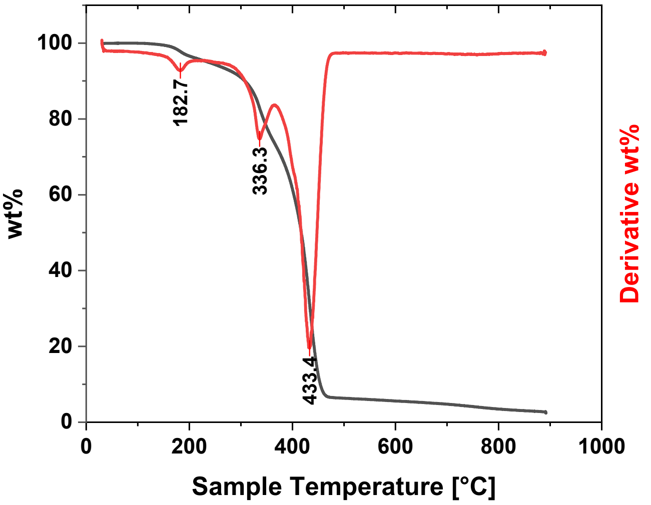

Supplementary Figure S25. *TGA measurement from room temperature till 900 °C for a 75% PS-TAAD material under* N_2.

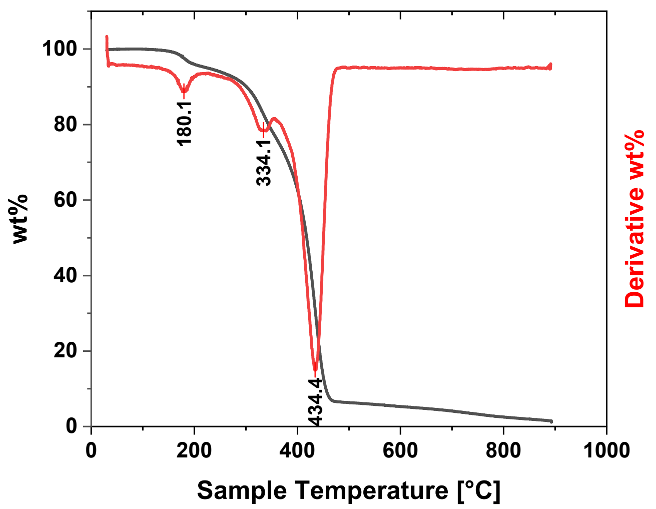

Supplementary Figure S26. *TGA measurement from room temperature till 900 °C for a 50% PS-TAAD material under N_2.*

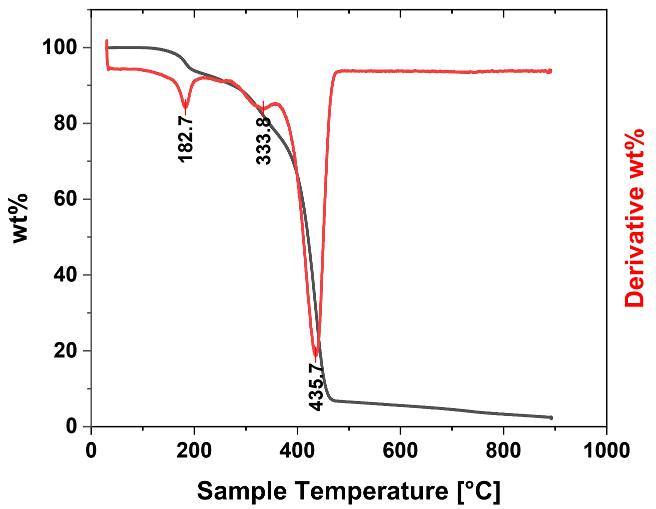

Supplementary Figure S27. *TGA measurement from room temperature till 900 °C for a 25% PS-TAAD material under N_2.*

Solvent study data

This next section contains the raw weight data for the solvent study with the different crosslinking degrees.

Supplementary Table S3. *Swelling and solvation data of a 100% crosslinked PS-TAAD (batch 1).*

solvent	weighted-in (mg)	wet weight (mg)	dry weight (mg)
**acetone**	3.4	5.9	3.3
**water**	4.8	5.2	4.5
**methanol**	5.2	10.2	4.8
**DCM**	5.4	28.8	5.2
**THF**	3.2	9.9	2.7
**DMF**	7.4	35.4	7.2

Supplementary Table S4. *Swelling and solvation data of a 100% crosslinked PS-TAAD (batch 2).*

solvent	weighted-in (mg)	wet weight (mg)	dry weight (mg)
**acetone**	5.7	9.5	5.4
**water**	5.3	6.6	5.1
**methanol**	4.7	9.5	4.3
**DCM**	8.1	41.2	7.8
**THF**	7.8	26	7.8
**DMF**	8.1	33.7	7.8

Supplementary Table S5. *Swelling and solvation data of a 75% crosslinked PS-TAAD (batch 1).*

solvent	weighted-in (mg)	wet weight (mg)	dry weight (mg)
**acetone**	2.5	4.5	2.5
**water**	7.9	8.9	7.9
**methanol**	5.2	10.2	5.0
**DCM**	7.5	35.3	7.3
**THF**	9.2	28.5	8.8
**DMF**	5.9	26	5.9

Supplementary Table S6. *Swelling and solvation data of a 75% crosslinked PS-TAAD (batch 2).*

solvent	weighted-in (mg)	wet weight (mg)	dry weight (mg)
**acetone**	6.2	10.2	6.0
**water**	7.2	7.5	6.6
**methanol**	4.6	8.5	4.5
**DCM**	5	22.9	4.9
**THF**	5.4	17.2	5.4
**DMF**	6.9	29.1	6.7

Supplementary Table S7. *Swelling and solvation data of a 50% crosslinked PS-TAAD (batch 1).*

solvent	weighted-in (mg)	wet weight (mg)	dry weight (mg)
**acetone**	6.9	11.2	6.5
**water**	4	4.5	3.7
**methanol**	3.6	6	2.9
**DCM**	2.8	12.7	2.6
**THF**	4.3	15.5	4.3
**DMF**	5.7	32.8	5.4

Supplementary Table S8. *Swelling and solvation data of a 50% crosslinked PS-TAAD (batch 2).*

solvent	weighted-in (mg)	wet weight (mg)	dry weight (mg)
**acetone**	3.4	6.1	3.3
**water**	7.5	8.7	7.4
**methanol**	7.1	13.4	6.5
**DCM**	6.3	33.7	6.2
**THF**	9.7	35	9.7
**DMF**	6.9	31	6.2

Supplementary Table S9. *Swelling and solvation data of a 25% crosslinked PS-TAAD (batch 1).*

solvent	weighted-in (mg)	wet weight (mg)	dry weight (mg)
**acetone**	3.9	5.8	3.6
**water**	6.8	7.3	6.4
**methanol**	6.1	14.6	5.6
**DCM**	4.7	18.5	4.6
**THF**	6	18.7	5.9
**DMF**	6.5	78.4	6.0

Supplementary Table S10. *Swelling and solvation data of a 25% crosslinked PS-TAAD (batch 2).*

solvent	weighted-in (mg)	wet weight (mg)	dry weight (mg)
**acetone**	4.9	7.1	4.9
**water**	3.3	8.6	3.0
**methanol**	2.6	5	2.4
**DCM**	4.6	24.3	4.6
**THF**	3.1	17	3.1
**DMF**	4.1	55.3	3.4

Short PS-TAAD polymer materials methods and data

5.2 mL (45 mmol) of styrene (after inhibitor removal via basic aluminum oxide column) and 0.99 g (5.0 mmol) of 4-vinylbenzyl bromide (73% Br/ 27% Cl) were dissolved in 7.5 mL anhydrous toluene in a 25 mL round bottom flask. 1.45 mL (0.27 mmol) of 0.2 M AIBN in toluene and 71.7 mg (0.198 mmol) of 4-Cyano-4-[(dodecylsulfanylthiocarbonyl)sulfanyl]pentanoic acid were added. The flask was then sealed with a septum and parafilm. The mixture was then cooled in an ice bath and purged with N_2 for 30 minutes. After purging the flask was sealed and the reaction mixture was heated at 65 °C overnight. The reaction was quenched by introduction of oxygen and the viscous liquid was diluted with 5 mL THF. The polymer was then precipitated into ice cold methanol 2 times. After solvent evaporation the polymer was dried in a vacuum oven overnight at 50 °C, resulting in 1.6 g product.

Supplementary Table S11. *Characterization of the obtained low molecular weight poly(styrene-co-4-vinylbenzyl bromide).*

M_n (g/mol)	7050
M_w (g/mol)	9550
Dispersity	1.4
Chain length	62
4-vinylbenzyl bromide/chloride in chain%	13%
4-vinyl benzyl/ chain	8

To functionalize low molecular weight poly(styrene-co-4-vinylbenzyl bromide), 1.56 g (0.21 mmol) polymer was reacted with 0.40 g (1.7 mmol) $TRISOXH_3$ in 30 mL THF : MeOH (1 : 1) over 7 days at room temperature. After the reaction, the product was precipitated twice in diethyl ether and dried overnight in a vacuum oven at 50 °C. This resulted in 1.12 g product. NMR showed roughly 80% functionalization.

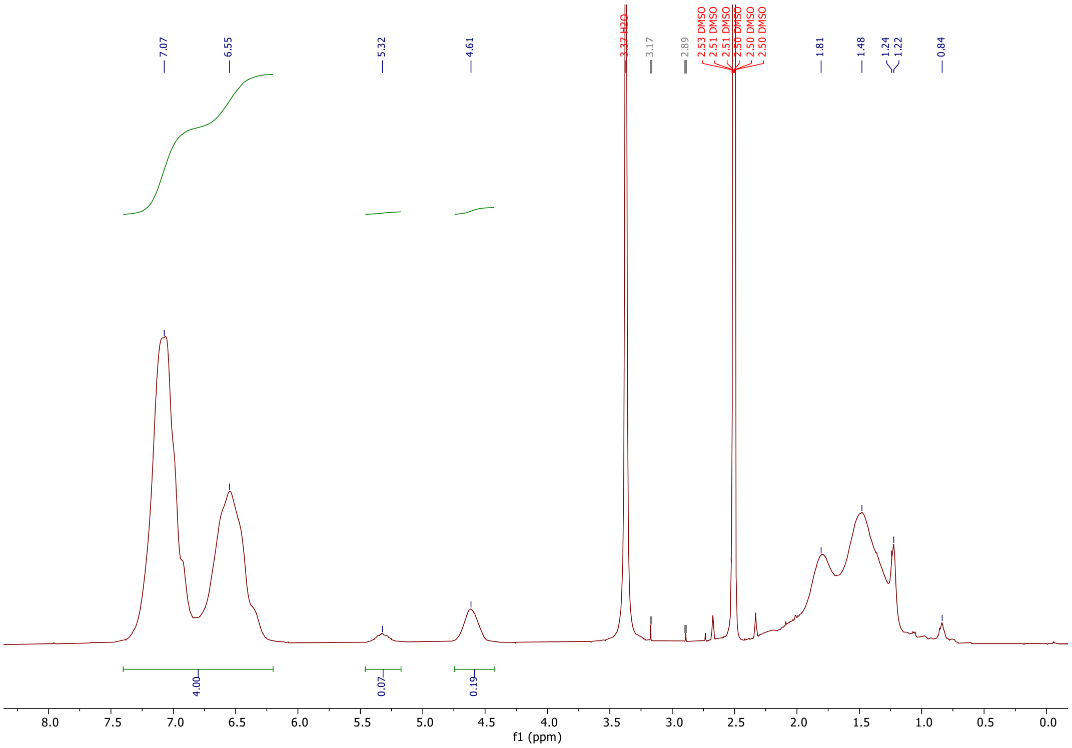

Supplementary Figure S28. *^{1}H-NMR of low molecular weight polystyrene-co-4-vinylbenzyl bromide in d6-DMSO.*

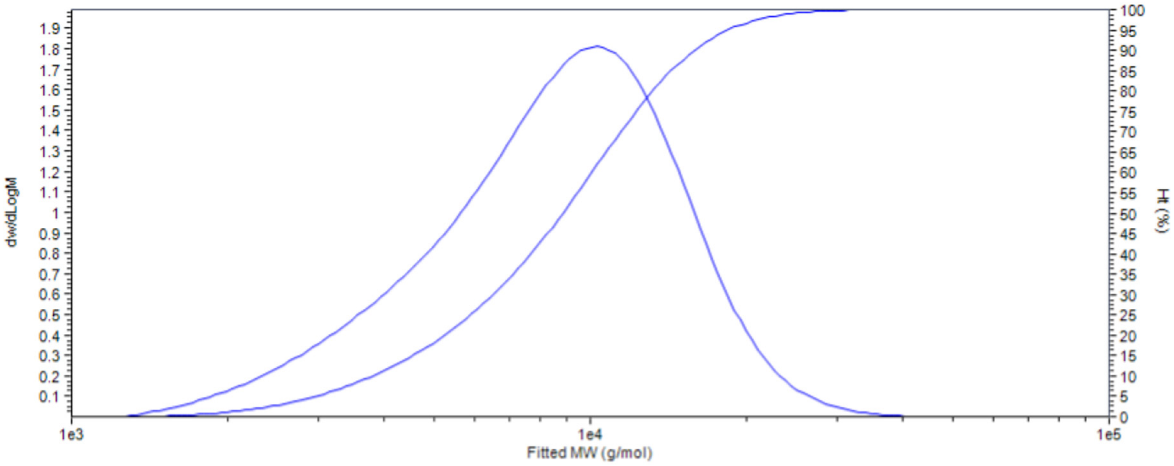

Supplementary Figure S29. *GPC trace of low molecular weight polystyrene-co-4-vinylbenzyl bromide in THF.*

RT (min)	M_n (g/mol)	M_w (g/mol)	Dispersity
8.336	7044	9561	1.4

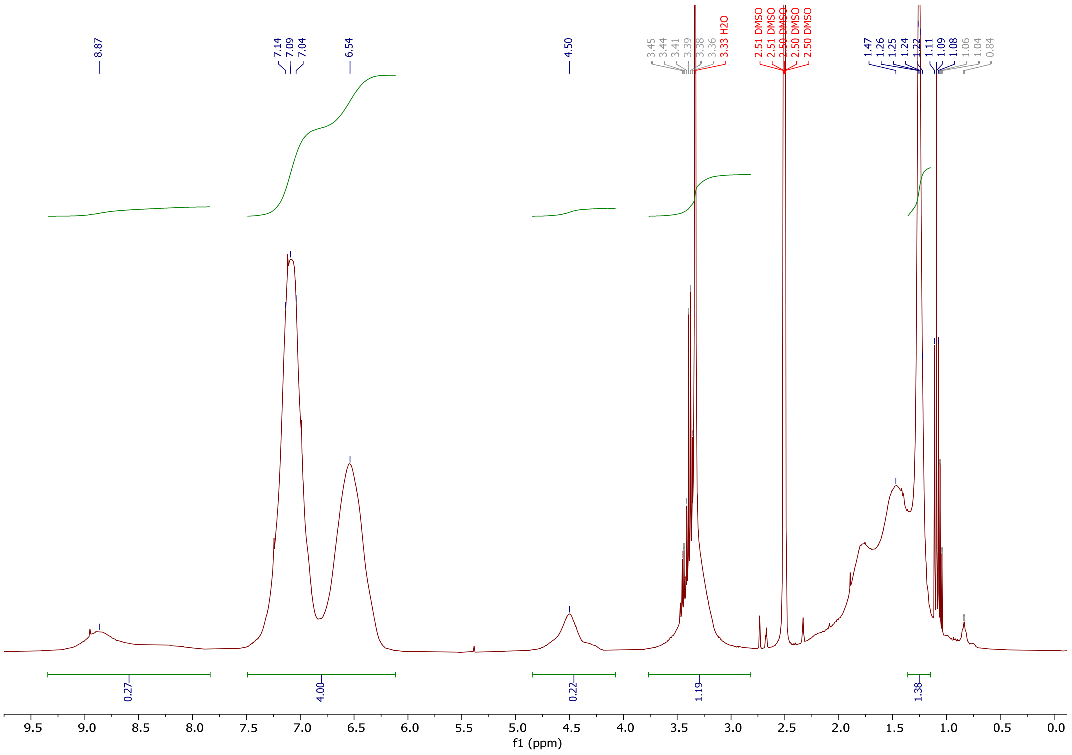

Supplementary Figure S30. *^{1}H-NMR of TAAD functionalized low molecular weight polystyrene-co-4-vinylbenzyl bromide in d_6-DMSO.*

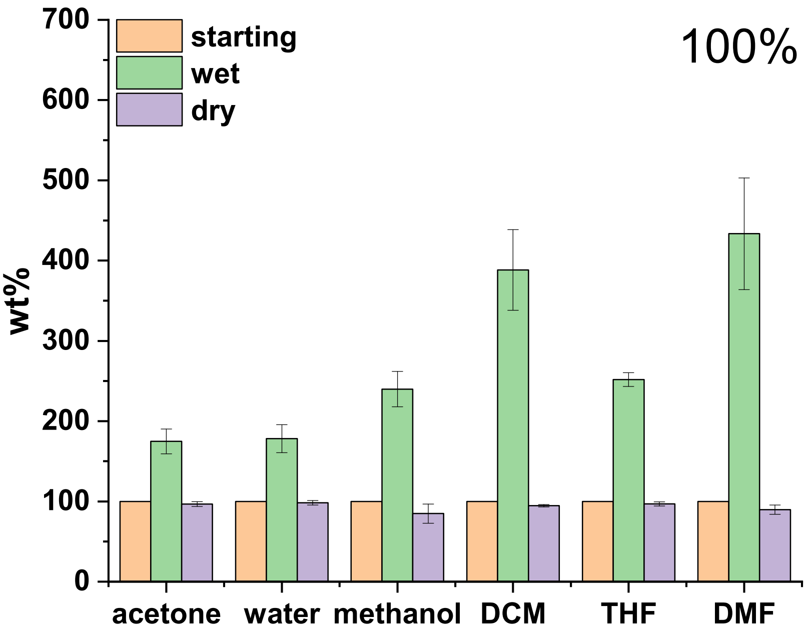

Supplementary Figure S31. *Solvent study of the short TAAD functionalized polystyrene materials with 100% crosslinking. All measurements were performed in triplo.*

Supplementary Table S12. *Swelling and solvation data of the 100% crosslinked materials with the short TAAD functionalized polystyrene polymers (batch 1).*

solvent	weighted-in (mg)	wet weight (mg)	dry weight (mg)
**acetone**	8.1	15.6	7.8
**water**	6.2	10.8	5.9
**methanol**	8.8	23.3	6.4
**DCM**	5.6	18.7	5.2
**THF**	7.2	17.7	6.9
**DMF**	8.4	33	7.1

Supplementary Table S13. *Swelling and solvation data of the 100% crosslinked materials with the short TAAD functionalized polystyrene polymers (batch 2).*

solvent	weighted-in (mg)	wet weight (mg)	dry weight (mg)
**acetone**	7.6	12.7	7.6
**water**	6.5	10.6	6.5
**methanol**	5.9	13.7	5.7
**DCM**	6.4	27.7	6.1
**THF**	9.8	24.3	9.3
**DMF**	9.6	37.8	8.5

Supplementary Table S14. *Swelling and solvation data of the 100% crosslinked materials with the short TAAD functionalized polystyrene polymers (batch 3).*

solvent	weighted-in (mg)	wet weight (mg)	dry weight (mg)
**acetone**	8.2	13.5	7.7
**water**	7.4	14.6	7.4
**methanol**	6.1	13.6	5.2
**DCM**	11.7	46.6	11.2
**THF**	5.2	13.6	5.2
**DMF**	5.1	26.2	4.9

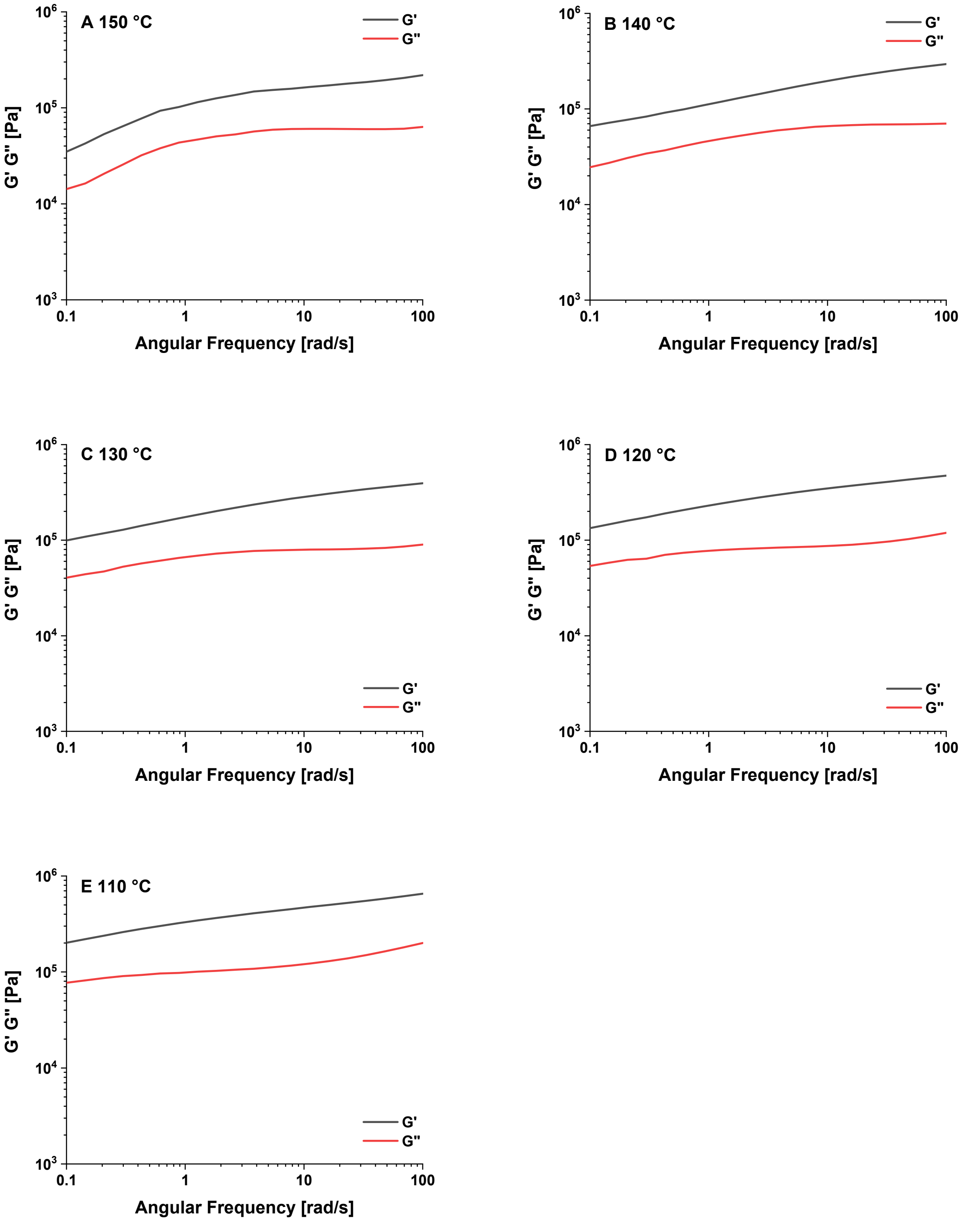

Supplementary Figure S32. *Frequency sweeps of the 100% crosslinked materials with the short TAAD functionalized polystyrene materials at various temperatures and 0.1% strain.*

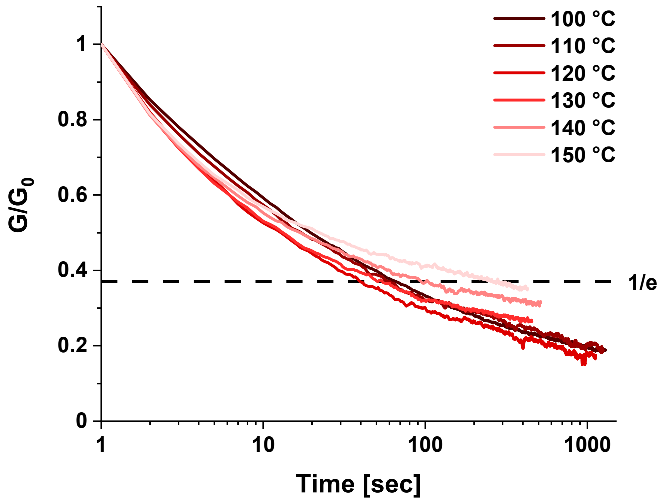

Supplementary Figure S33. *Relaxation data of the 100% crosslinked materials with the short TAAD functionalized polystyrene materials at 100 °C and 0.1% strain.*

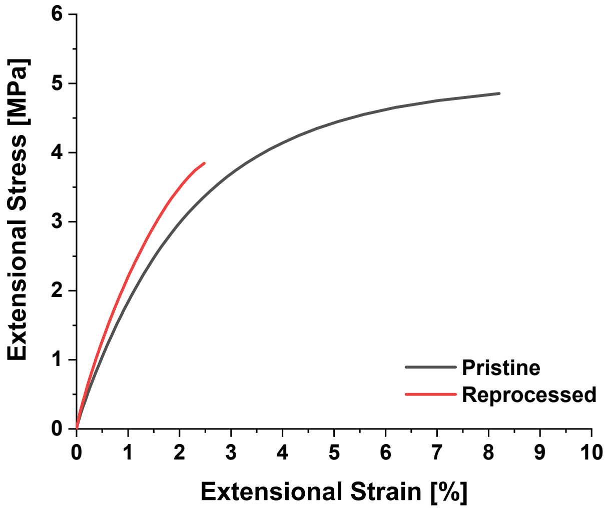

Supplementary Figure S34. *Extensional DMA measurements on the pristine and recycled short TAAD functionalized polystyrene material.*

PTSA addition (10 wt%)

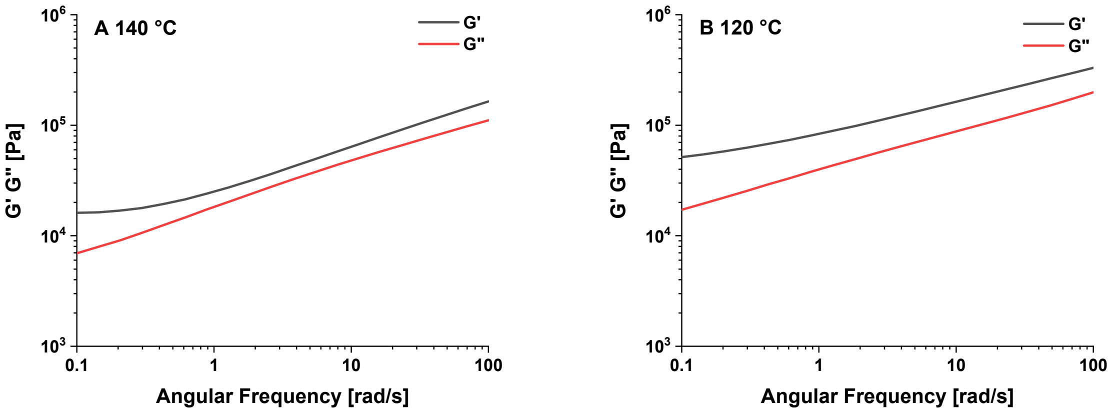

Supplementary Figure S35. *Frequency sweep of the 100% crosslinked materials with the short TAAD functionalized polystyrene materials and 10wt% PTSA at 120 and 140 °C with 0.1% strain.*

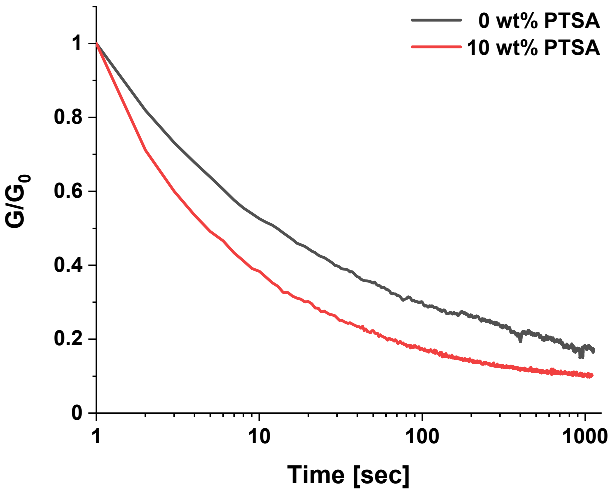

Supplementary Figure S36. *Relaxation curves of the 100% crosslinked materials with the short TAAD functionalized polystyrene materials 0 wt% or 10 wt% PTSA at 120 °C and 0.1% strain.*

Polystyrene reference (97 kDa) data

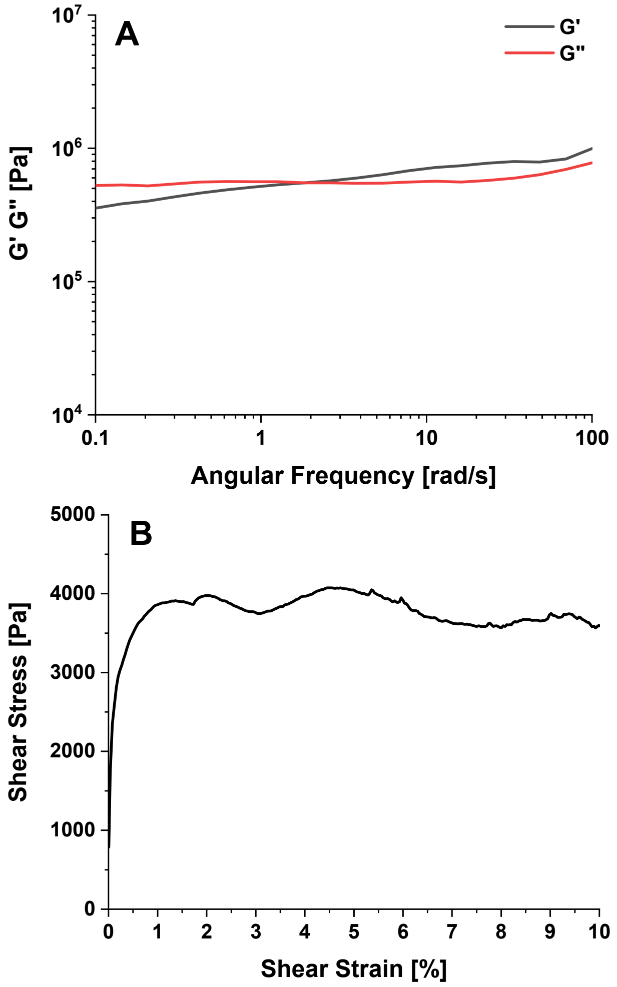

Supplementary Figure S37. *Frequency sweep of the polystyrene reference polymer (97 kDa) with 0.1% strain at 100 °C (A). Shear experiment of the polystyrene reference polymer (97 kDa) at 100 °C (B).*

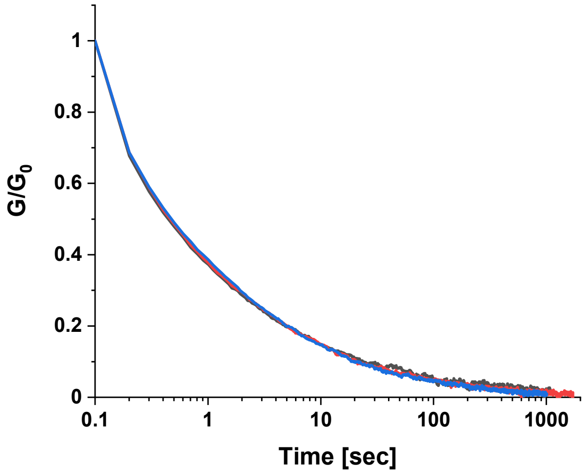

Supplementary Figure S38. *Relaxation curves of the polystyrene reference polymer (97 kDa) with 0.1% strain at 100 °C. n = 3.*

Photos reprocessing

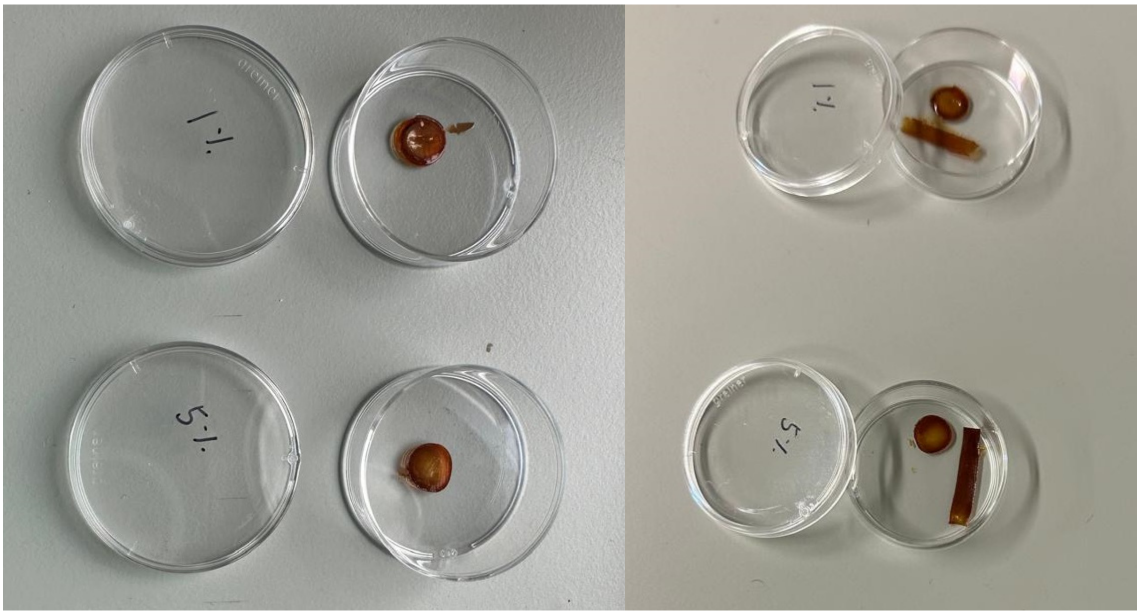

Supplementary Figure S39. *Photos taken of PS-TAAD materials with 100% crosslinking and 1 or 5 wt% PTSA before (left) and after (right) reprocessing.*

References

S1. Golovanov, I. S.; Mazeina, G. S.; Nelyubina, Y. V.; Novikov, R. A.; Mazur, A. S.; Britvin, S. N.; Tartakovsky, V. A.; Ioffe, S. L.; Sukhorukov, A. Y., Exploiting Coupling of Boronic Acids with Triols for a pH-Dependent "Click-Declick" Chemistry. *J. Org. Chem.* **2018,** *83* (17), 9756-9773.

S2. Chakma, P.; Digby, Z. A.; Shulman, M. P.; Kuhn, L. R.; Morley, C. N.; Sparks, J. L.; Konkolewicz, D., Anilinium Salts in Polymer Networks for Materials with Mechanical Stability and Mild Thermally Induced Dynamic Properties. *ACS Macro Lett* **2019,** *8* (2), 95-100.

S3. Bao, C. Y.; Jiang, Y. J.; Zhang, H. Y.; Lu, X. Y.; Sun, J. Q., Room-Temperature Self-Healing and Recyclable Tough Polymer Composites Using Nitrogen-Coordinated Boroxines. *Adv. Funct. Mater.* **2018,** *28* (23), 1800560.
